# LYN kinase programs stromal fibroblasts to facilitate leukemic survival via regulation of c-JUN and THBS1

**DOI:** 10.1038/s41467-023-36824-2

**Published:** 2023-03-10

**Authors:** Alexander F. vom Stein, Rocio Rebollido-Rios, Anna Lukas, Maximilian Koch, Anton von Lom, Sebastian Reinartz, Daniel Bachurski, France Rose, Katarzyna Bozek, Ali T. Abdallah, Viktoria Kohlhas, Julia Saggau, Rebekka Zölzer, Yue Zhao, Christiane Bruns, Paul J. Bröckelmann, Philipp Lohneis, Reinhard Büttner, Björn Häupl, Thomas Oellerich, Phuong-Hien Nguyen, Michael Hallek

**Affiliations:** 1 University of Cologne, Faculty of Medicine and University Hospital Cologne, Department I of Internal Medicine, Center for Integrated Oncology Aachen Bonn Cologne Duesseldorf, Cologne, Germany; 2 Center for Molecular Medicine Cologne, University of Cologne, Cologne, Germany; 3 CECAD Center of Excellence on Cellular Stress Responses in Aging-Associated Diseases, University of Cologne, Cologne, Germany; 4 Mildred Scheel School of Oncology Aachen Bonn Cologne Düsseldorf, Faculty of Medicine and University Hospital of Cologne, Cologne, Germany; 5 University of Cologne, Institute for Biomedical Informatics, Faculty of Medicine and University Hospital Cologne, Cologne, Germany; 6 Faculty of Medicine and University Hospital Cologne, Department of General, Visceral and Cancer Surgery, University of Cologne, Cologne, Germany; 7 Max-Planck Institute for the Biology of Ageing, Cologne, Germany; 8 Reference Centre for Lymph Node Pathology and Hematopathology, Hämatopathologie Lübeck, Lübeck, Germany; 9 Faculty of Medicine and University Hospital Cologne, Department of Pathology, University of Cologne, Cologne, Germany; 10 Department of Hematology/Oncology, Johann Wolfgang Goethe University, Frankfurt, Germany; 11 German Cancer Consortium (DKTK), Heidelberg, Germany; 12 German Cancer Research Center (DKFZ), Heidelberg, Germany; 13 Frankfurt Cancer Institute, Goethe University Frankfurt, Frankfurt, Germany

**Keywords:** Cancer microenvironment, Chronic lymphocytic leukaemia

## Abstract

Microenvironmental bystander cells are essential for the progression of chronic lymphocytic leukemia (CLL). We have discovered previously that LYN kinase promotes the formation of a microenvironmental niche for CLL. Here we provide mechanistic evidence that LYN regulates the polarization of stromal fibroblasts to support leukemic progression. LYN is overexpressed in fibroblasts of lymph nodes of CLL patients. LYN-deficient stromal cells reduce CLL growth in vivo. LYN-deficient fibroblasts show markedly reduced leukemia feeding capacity in vitro. Multi-omics profiling reveals that LYN regulates the polarization of fibroblasts towards an inflammatory cancer-associated phenotype through modulation of cytokine secretion and extracellular matrix composition. Mechanistically, LYN deletion reduces inflammatory signaling including reduction of c-JUN expression, which in turn augments the expression of Thrombospondin-1, which binds to CD47 thereby impairing CLL viability. Together, our findings suggest that LYN is essential for rewiring fibroblasts towards a leukemia-supportive phenotype.

## Abstract (web summary)

The survival of chronic lymphocytic leukemia cells strongly depends on the presence of a supportive microenvironment. Here, the authors show that LYN kinase is essential for the reprogramming of stromal cells towards a leukemia-supportive phenotype that facilitates disease progression.

## Introduction

The pathobiology of chronic lymphocytic leukemia (CLL) is characterized by the accumulation of long-lived malignant B cells in the peripheral blood and hemato-lymphoid tissues, where leukemic cells interact reciprocally with various cells of the microenvironment^1^. The efficient dialog with diverse microenvironmental factors is crucial for CLL cell survival and expansion^2,3^. In this context, bone marrow stromal cells (BMSC) contribute to the interdependent generation of a nurturing niche and promote leukemic cell migration, proliferation and chemoresistance via various signaling pathways^4^. Leukemic cells activate inflammatory pathways in BMSC, including AKT, ERK, PKCβII and NFκB pathways, which trigger an increased expression of inflammatory and wound healing associated proteins, ultimately resulting in an increased leukemia support^5,6^. The molecular signature and phenotype of CLL-induced BMSC resemble those of cancer-associated fibroblasts (CAF), a diverse group of activated fibroblasts, primarily observed in association with many solid cancers^7,8^.

CAF are heterogenous in regard to their cellular origin, activation, phenotype and function and may diverge within and between entities^7^. Therefore, the precise characterization of CAF via cellular markers remains a challenge. Single-cell analyses have consistently identified an IL1-JAK-STAT induced inflammatory “iCAF” subtype that supports tumor growth via secretion of variable cytokines, and a TGFβ-induced myofibroblastic “myCAF” subtype with potential tumor-restraining functions via the extracellular matrix (ECM)^9^. In addition, other subtypes like immunomodulatory CAF showing an increased interferon signature and MHC expression linked with antigen presenting functions^10,11^ highlight the capacity of CAF to shape non-malignant components of the tumor-microenvironment.

The existence and functional relevance of stromal cells that partially reflect a CAF phenotype has been described in various hematological malignancies^12–18^, suggesting a generic importance of leukemia/lymphoma associated fibroblasts for disease development. In CLL, the upregulation of the standard CAF marker αSMA was observed in the lymph node microenvironment in vivo and in vitro^5,6^.

We previously showed that the expression of the SRC family kinase (SFK) LYN was an essential factor for CLL progression in vivo as demonstrated by adoptive transfer experiments in the *Eµ-TCL1* transgenic mouse model for CLL^19^, which could be partially attributed to the failure of LYN-deficient macrophages to fully support CLL cell survival^20^. Although LYN is well-known to balance the B cell receptor (BCR) response, activation of this kinase seemed to play only a minor role in B-CLL development, as loss- and gain-of-function mutants of LYN in B cells did not significantly alter CLL progression in murine models^2,20,21^. In contrast, LYN expression in the microenvironment was essential for leukemic cell growth, which could be partially attributed to the failure of LYN-deficient macrophages to fully support CLL cell survival^20^. Interestingly, the specific loss of LYN in the hematopoietic system did not allow to fully explain the impaired CLL development observed in the global absence of microenvironmental LYN. Therefore, we hypothesized that the presence of LYN kinase in non-hematopoietic compartments of the leukemic stroma may substantially contribute to leukemic progression.

In this work, we demonstrate that stromal LYN kinase modulates the plasticity of stromal fibroblasts and their capacity to support the survival of malignant B cells. Furthermore, we identify the LYN-cJUN-THBS1 axis as a key mediator in this process. This finding underscores the specific importance of LYN kinase as a central regulator in the tumor microenvironment.

## Results

### LYN kinase in non-hematopoietic microenvironmental cells promoted CLL expansion and survival

**Fig. 1:**
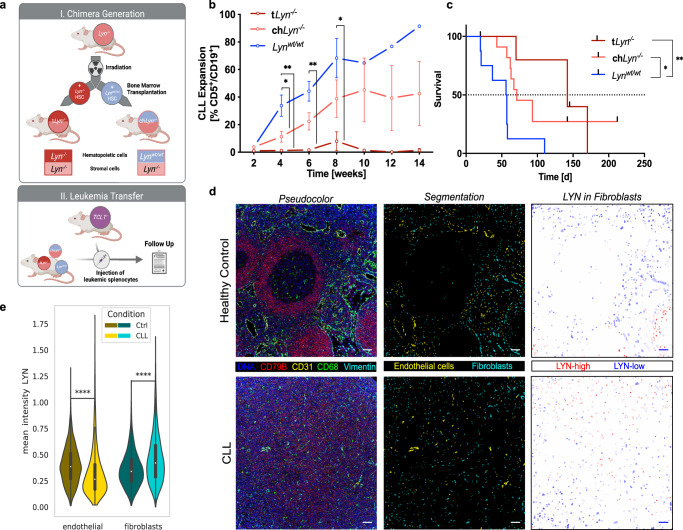
LYN kinase in non-hematopoietic microenvironmental cells promoted CLL expansion. **a** Graphical illustration of the in vivo experimental setup. I. *Lyn*^*−/−*^ mice were lethally irradiated and the hematopoietic system was reconstituted using *Lyn*^*−/−*^ or *Lyn*^*wt/wt*^ bone marrow transplantation (BMT), resulting in chimeric mice lacking LYN globally (t*Lyn*^*−/−*^) or specifically in the radioresistant stromal compartment (ch*Lyn*^*-/-*^). II. The hematopoietic system was successfully reconstituted after 8 weeks and chimeric mice were subsequently transplanted with 10^7^ murine *TCL1*^*tg/w*t^ CLL cells (4 biological replicates). Illustration was created with BioRender.com. **b** Leukemic expansion in the peripheral blood was monitored every 14 days via flow cytometry and is displayed as fraction of CD5^+^CD19^+^ CLL cells in all CD45^+^ leukocytes (t*Lyn*^*−/−*^ *n* = 7; ch*Lyn*^*−/−*^ *n* = 10; *Lyn*^*wt/wt*^ *n* = 8; mean ± SEM; two-sided Mann-Whitney test: Week 4 *Lyn*^*wt/wt*^ vs. *chLyn*^*−/−*^ *p* = 0.02499, Week 4 *Lyn*^*wt/wt*^ vs. *tLyn*^*−/−*^ *p* = 0.001166, Week 6 *Lyn*^*wt/wt*^ vs. *tLyn*^*−/−*^ *p* = 0.002165, Week 8 *Lyn*^*wt/wt*^ vs. *tLyn*^*−/−*^ *p* = 0.035714). **c** Kaplan-Meier curve illustrating the survival of chimeric recipient mice after CLL adoptive transfer (t*Lyn*^*−/−*^ *n* = 6; ch*Lyn*^*−/−*^ *n* = 11; *Lyn*^*wt/wt*^ *n* = 8; Mantel-Cox log-rank test: *Lyn*^*wt/wt*^ vs. *chLyn*^*−/−*^ *p* = 0.0119, *Lyn*^*wt/wt*^ vs. *tLyn*^*−/−*^ *p* = 0.0021*, chLyn*^*−/−*^ vs. *tLyn*^*−/−*^ *p* = 0.4707). **d** Representative images of Hyperion Imaging Mass Cytometry of a healthy control lymph node (HC-LN) (*top*) and a CLL-LN (*bottom*). (*Left*) False color image (*middle*) result of cell-type segmentation (*right*) segmented fibroblasts color-coded by mean LYN intensity (bars represent 50 µm, representative of total *n* = 9 HC-LN and *n* = 12 CLL-LN). **e** Violin plot for mean LYN expression per segmented single cell (Healthy controls: *n* = 9 HC-LN (3272 endothelial cells; 6466 fibroblasts); CLL: *n* = 12 CLL-LN (1817 endothelial cells; 7543 fibroblasts); boxplots represent median with the interquartile range, whiskers indicate adjacent values, violin represents kernel density estimation, independent two-sided t-tests: endothelial *p* = 7.380e−41, fibroblasts *p* = 2.942e−170). See also Fig. S1. Source data are provided as a Source Data file.

To assess the potential relevance of LYN kinase in the stromal compartment in vivo, we generated chimeric mice lacking LYN specifically in non-hematopoietic cells via bone marrow transplantation (BMT) of *Lyn*^−/−^ host mice. After receiving a lethal dose of whole-body irradiation, the hematopoietic system of irradiated mice was reconstituted using hematopoietic stem cells (HSC) from either wild type (WT) or *Lyn*^−/−^ donors, yielding either totally deficient *Lyn* mice (t*Lyn*^−/−^) or chimeric mice lacking *Lyn* only in the radioresistant, non-hematopoietic microenvironment (ch*Lyn*^*−/−*^), respectively (Fig. 1a). After successful immune-reconstitution, t*Lyn*^*−/−*^ mice showed an expanded myeloid fraction and a complete loss of B cells—a phenotype typically observed in aged *Lyn*^*−/−*^ mice−, whereas ch*Lyn*^*−/−*^ mice acquired an increased fraction of B cells and normalized myeloid compartment (Supplementary Fig. 1a). Murine CLL cells purified from spleens of *Eµ-TCL1* transgenic mice (*TCL1*^+^)^19^ were subsequently transferred onto the chimeras (Fig. 1a). Expansion of CD5^+^/CD19^+^ CLL cells could be detected in the peripheral blood after 4 weeks in *Lyn*^*wt*/wt^ and ch*Lyn*^*−/−*^ mice, but not in t*Lyn*^*−/−*^ mice (Fig. 1b), reproducing the failure of leukemic engraftment observed before in aged global *Lyn*^*−/−*^ mice^20^. Notably, the leukemic burden was significantly lower in ch*Lyn*^-/-^ mice in comparison to *Lyn*^wt/wt^ mice in week 4 and remained consistently lower until week 14 (Fig. 1b), indicating that LYN-dependent functions within the non-hematopoietic microenvironment contributed to the generation of a fully leukemia supporting niche. The failure of CLL cell migration into the lymphoid tissues in *Lyn*^*−/−*^ recipients could be excluded, because CLL cells could efficiently home to the spleen and bone marrow of both *Lyn*^*wt*/wt^ and *Lyn*^*−/−*^ recipients in an in vivo short-term homing assay (Supplementary Fig. 1b). In concordance, the reduced leukemic burden in ch*Lyn*^*−/−*^ mice resulted in a significantly prolonged median overall survival of 71 days versus 56 days in *Lyn*^wt/wt^ mice (*p* = 0.002) (Fig. 1c), collectively demonstrating a relevant, leukemia promoting function of LYN kinase in the non-hematopoietic microenvironment.

To investigate the expression of LYN kinase in the non-hematopoietic microenvironment of CLL patients, we used imaging mass cytometry to compare lymph node sections of 12 CLL patients (CLL-LN) and 9 healthy controls (HC-LN). Representative CLL-LN showed the typical disappearance of the LN architecture compared to HC-LN, with CLL-LN follicles being disrupted by the densely infiltrating leukemic B cells, accompanied by a widely scattered distribution of different bystander cells (Fig. 1d*left*). Single cells were segmented and stromal cells assigned into endothelial cells (CD31^+^) and fibroblasts (CD31^−^ Vimentin^+^) (Fig. 1d*middle*). Strikingly, CLL-LN fibroblasts exhibited significantly increased LYN levels compared to HC-LN fibroblasts, whereas CLL-LN endothelial cells showed markedly lower LYN expression than HC-LN endothelial cells (Fig. 1e). These findings point to a cell-type specific modulation of LYN expression in CLL lymph nodes and to a pathophysiological relevance of LYN in CLL-associated fibroblasts.

**Fig. 2:**
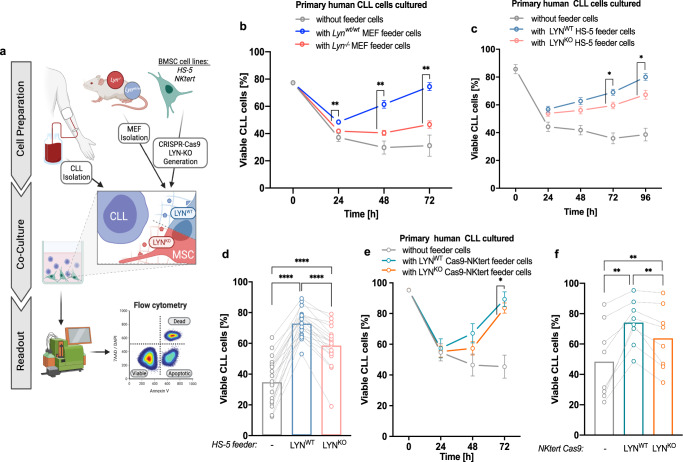
LYN kinase in fibroblasts supported primary CLL cell survival in vitro. **a**, Illustration of the different in vitro co-culture systems: Primary human CLL samples were isolated from the peripheral blood of CLL patients. LYN proficient and deficient stromal feeder cells were either isolated from MEF of *Lyn*^*wt/wt*^ and *Lyn*^*−/−*^ mice or generated in human BMSC lines via CRISPR/Cas9. Leukemic and stromal cells were co-cultured, and CLL viability (defined as proportion of Annexin V^-^/7AAD^−^ cells of all CD45^+^ cells) was measured over time by flow cytometry. Illustration was created with BioRender.com. **b** *Lyn*^*wt/wt*^ and *Lyn*^*−/−*^ MEF (*n* = 2 clones per genotype) were used in co-culture with primary human CLL samples (*n* = 3). Depicted is the CLL viability over time (mean ± SEM; two-sided Mann-Whitney test for *Lyn*^*wt/wt*^ vs. *Lyn*^*−/−*^ at each time point: 24 h–72 h *p* = 0.002165). **c**, **d** LYN^WT^ and LYN^KO^ HS-5 single-cell clones (SCC) were used in co-culture with CLL cells. (CLL *n* = 19; 2 clones per HS-5 genotype). **c** illustrates CLL viability over time (mean ± SEM; two-sided Mann-Whitney test for LYN^WT^ vs. LYN^KO^ at each time point: 72 h *p* = 0.003056; 96 h *p* = 0.013878) and (**d**) highlights individual sample viability after 72 h of co-culture (single representative HS-5 clone per genotype; bars represent mean viability; two-tailed Wilcoxon rank test: in all tests *p* < 0.0001). **e**, **f** LYN^WT^ and LYN^KO^ NKtert Cas9 SCCs were used for co-culture assays with CLL cells. (CLL *n* = 10; LYN^WT^ 1 clone, LYN^KO^ 5 clones). **e** illustrates CLL viability over time (mean ± SEM; two-sided Mann-Whitney test for LYN^WT^ vs. LYN^KO^ at each time point: 72 h *p* = 0.049609) and (**f**) highlights individual sample viability after 48 h of co-culture (from 8 CLL patients; LYN^WT^: 1 clone; LYN^KO^: every circle represents mean viability on 5 distinct LYN^KO^ clones; bars represent mean viability; Wilcoxon rank test: in all tests *p* = 0.0078). See also Fig. S1. Source data are provided as a Source Data file.

As fibroblasts represent one of the most potent non-immune supporter cell types for cancer growth^7,8^ and can prevent spontaneous apoptosis of CLL cells^4^, we used murine embryonic fibroblasts (MEF) in a xenogeneic co-culture system with human CLL cells (Fig. 2a). We observed a strongly reduced feeding capacity of *Lyn*^*−/−*^ MEF to maintain CLL cell survival compared to *Lyn*^*wt/wt*^ cells (Fig. 2b, Supplementary Fig. 1c), further underscoring the functional relevance of LYN kinase in the stromal microenvironment. To recapitulate the murine results in a fully human co-culture setting in vitro, we generated *LYN*-knockout (LYN^KO^) single-cell clones of the human bone marrow derived stromal cell line HS-5 (Supplementary Fig. 1d, 1j i) by CRISPR/Cas9-induced frameshift mutation (Fig. S1e), which did not affect HS-5 cell growth (Supplementary Fig. 1f). Similar to MEF, LYN^KO^ HS-5 cells could not support CLL cell survival as potently as LYN^WT^ control cells in co-culture, revealed by a significantly reduced CLL viability after 72 h and 96 h (Fig. 2c). We predominantly used low-risk, treatment naïve patient samples (Supplementary Fig. 1g) with a strongly variable rate of spontaneous cell death, ranging from 12% to 60% viability after 72 h of culture. Still, intra-patient pairwise comparison demonstrated a highly consistent reduction of HS-5 feeder support in the absence of LYN (Fig. 2d) and independently of the IGVH status of CLL samples (Supplementary Fig. 1h). In addition, LYN^KO^ clones were generated in a second stromal cell line NKtert (Supplementary Fig. 1i, j ii). Like the HS-5 co-culture, LYN^KO^ NKtert cells also exhibited impaired leukemic feeder function compared to LYN^WT^ counterparts, revealed by a consistent reduction of CLL cell viability in co-culture (Fig. 2e, f). In marked contrast to fibroblasts, knockdown of LYN kinase in the HUVEC endothelial cell line did not influence their CLL supportive capacity (Supplementary Fig. 1k, l). Taken together, in vivo and in vitro results strongly indicate a functional role of LYN in stromal fibroblasts contributing to microenvironmental CLL support.

### Integrative multi-omics analysis identified a transcriptionally altered fibroblast polarization in LYN-deficient cells perturbating predominantly cytokine- and extracellular matrix related pathways

**Fig. 3:**
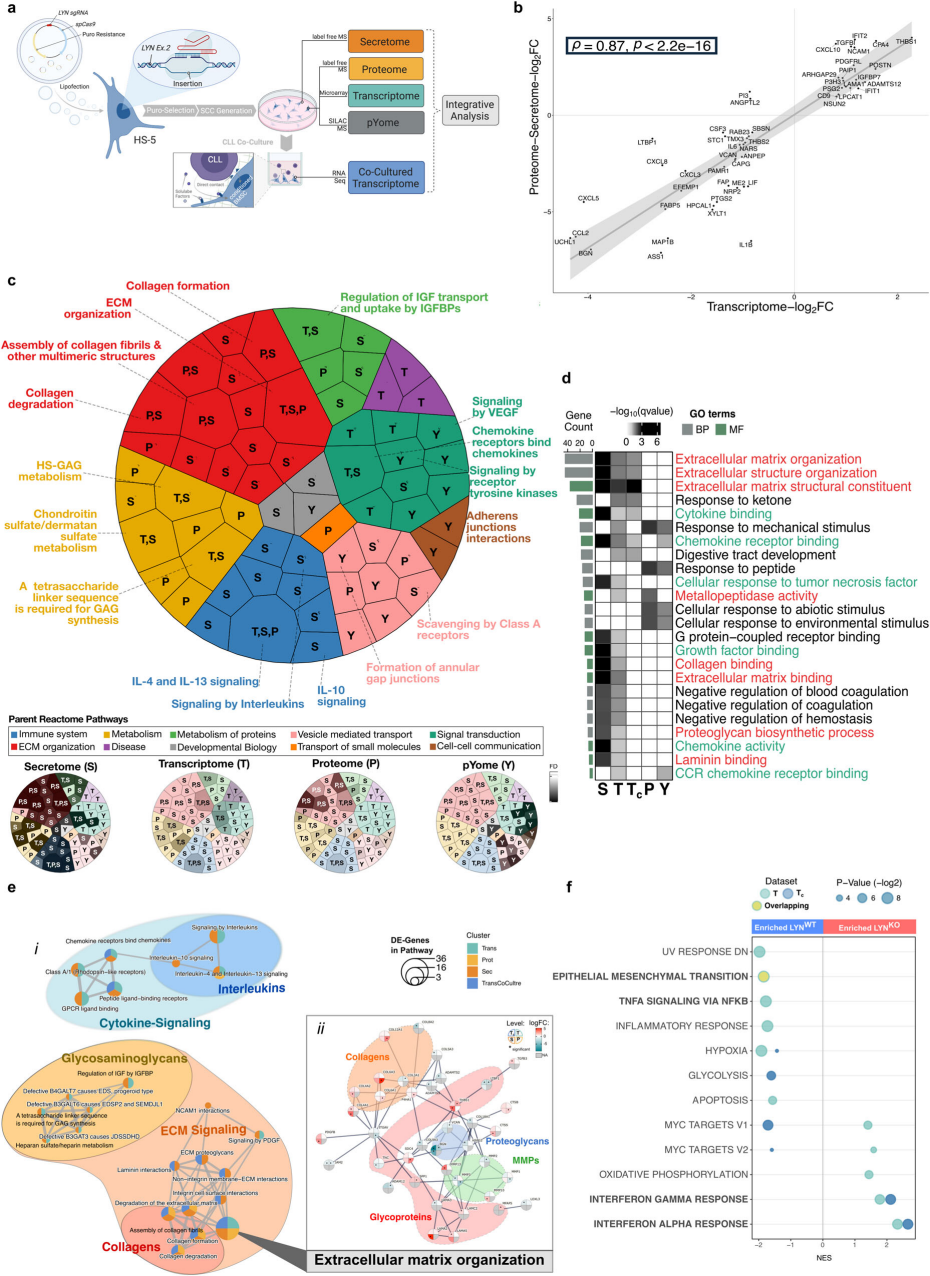
Integrative multi-omics analysis identified a transcriptionally altered fibroblast polarization in LYN-deficient cells perturbating predominantly cytokine- and matrix related pathways. **a** Multi-omics profiling. Illustration was created with BioRender.com. **b** Spearman rank correlation analysis between T and S/P. Commonly expressed genes/proteins (52 in total) with absolute log_2_-FC > 0.8 were selected. **c** Integrative Reactome-Pathway Enrichment analysis. Weighted Voronoi Treemap (*top*) represents a hierarchal visualization of enriched top-level pathways (defined by single colors) enclosing sub-level pathways with the annotation of each enriched “omics”-layer. The proportional area matches the frequency and weight of this region per total. Grayscale plots (*bottom*) show statistical significance (FDR calculated by c*lusterProfiler*^80^) for each sub-level pathway per “omics”-layer. **d** Heatmap of enriched GO-Terms independently performed per “omics”-layer, common terms displaying statistical significance (−log_10_ q value calculated by *clusterProfiler*^80^) are depicted. GO-terms are colored according to top-level pathways in (**c**). Bar plots represent total number of genes contributing to the enriched term (*green*: Molecular Function; *gray*: Biological Process). **e** Network visualization of Reactome-enrichment results: (i) Enrichment map of the top deregulated pathways in T, T_c_, P, S. Each node represents one Reactome-Term, the circle colors indicate “omics”-layers in which the pathway was deregulated, circle size represents DEG number within this term. Edges represent shared genes between nodes, generated by *clusterProfiler*^80^. Domain annotation was added manually. (ii) STRING protein association network contributing to “ECM organization” Reactome-Pathway. Every node is divided in four segments (representing T, T_c_, P, S) colored by the gene/protein log_2_-FC value. Gray color indicates missing values, asterisks indicate significant differentially expression in the respective layer (specified in Methods). Well-defined clusters of different ECM protein families were manually annotated. Only proteins with interaction score ≥ 0.9 are shown. **f** Gene Set Enrichment Analysis (GSEA) in T and T_c_. Normalized enrichment scores (NES) of enriched genesets from the Hallmark Molecular Signatures Database are depicted. Colors represent “omics”-layers, dot size represents statistical significance as -log_2_ of adjusted *p* value (specified in Methods) of enriched terms. Genesets with negative NES were enriched in LYN^WT^, positive NES enriched in LYN^KO^ clones. See also Fig. S2. Source data are provided as a Source Data file.

To characterize the molecular consequences caused by LYN deficiency in stromal fibroblasts, we subjected LYN^WT^ and LYN^KO^ HS-5 cells to multi-omics profiling comprising four layers: transcriptome (T), label-free quantitative proteome (P) and secretome (S), and SILAC-labeled phosphoproteome (phospho-tyrosine (pYome) (Y)). Additionally, we profiled LYN^WT^ and LYN^KO^ HS-5 transcriptome after CLL co-culture (T_c_). All “omics” layers were combined in a multi-step integrative analysis (Fig. 3a). The profiling layers covered 19,511 and 20,193 transcripts (T and T_c_), 1,836 and 314 proteins (P and S), and 122 phospho-tyrosine sites (Y). Altogether, 213 (T), 810 (T_c_), 109 (P), 37 (S) and 18 (Y) were differentially expressed (LYN^KO^/LYN^WT^ HS-5 cells) (Supplementary Fig. 2a). Results of independent analyses are listed in Supplementary Data 1, 2 and Supplementary Fig. 2a, b. Differentially expressed genes/proteins (DEG/P) are depicted in Supplementary Fig. 2c–g, and data are deposited in different EMBL-EBP databases (see Data availability statement).

Overall, results of the transcriptomic and proteomic analyses were highly similar regarding individual DEG/Ps and enriched functional pathways, implying that numerous changes of protein expression resulted from transcriptional alterations. We tested the strength of the relationship between DEG/Ps of the (T), (P) and (S) “omics” layers using a Spearman correlation analysis, and found a strong dependence (*ρ* = 0.87) (Fig. 3b), indicating that LYN-deletion perturbed the transcriptional regulation of stromal cells.

Accordingly, integrative interpretation of independent Pathway (Reactome) (Fig. 3c) and Gene Ontology (GO) (Fig. 3d) term enrichment analyses consistently identified two major transformations under LYN^KO^/LYN^WT^ condition: the most striking alterations were extracellular matrix (ECM)-related processes followed by cytokine-related ones. Network visualization highlights clustering of top enriched Reactome-Terms into these two domains (Fig. 3e), which could be exemplified by the consistent deregulation of extracellular matrix proteins (BGN, TGFBI, EFEMP1, THBS1) along with cytokines (CCL2, CXCL8) in mono-culture (Supplementary Fig. 2b).

ECM alterations globally integrated the highest number of target genes (Fig. 3c*red*, 3d, Supplementary Fig. 2i) and additionally changes in metabolic functions could be linked to ECM organization (Fig. 3c*yellow* and *light green*), indicating a widespread ECM remodeling affecting the biogenesis and composition of LYN^KO^ ECM. More specifically, network visualization revealed clustering of enriched ECM terms related to collagens, ECM signaling and glycosaminoglycan metabolism (Fig. 3ei). The consistently enriched central pathway “ECM organization” showed a predominant differential expression of collagens and metallopeptidases (Fig. 3eii), in agreement with results from GO-Term analyses (Fig. 3d).

Cytokine related alterations were represented by differential expression of CCL2, CXCL5, CXCL8, CXCL10, IL1 or IL6 on the molecular level (Fig. 3b, Supplementary Fig. 2c–e, g) and enriched terms such as “Cytokine binding“ and “Chemokine receptor binding” in GO-Term analyses (Fig. 3d), and “Signaling by Interleukins” in Reactome-Pathway analyses (Fig. 3c*blue*, ei). In co-cultured HS-5 cells (T_c_), alterations in “Senescence-Associated Secretory Phenotype” pathway also indicated the perturbation of cytokine secretion (Supplementary Fig. 2i). Moreover, several cytokine-signaling associated pathways (Fig. 3c*green*) as well as processes related with “Cellular responses” to different stimuli were prominently altered in Proteome and pYome, but not in Transcriptome and Secretome layers (Fig. 3c, d). This might reflect the impaired intracellular signaling cascades generated by LYN depletion as well as putatively point to a shifted autocrine response as secondary effect following the altered cytokine profile.

Gene Set Enrichment Analysis (GSEA), a different enrichment algorithm, taking into account also changes in gene expression below the significance threshold, validated the previous enrichment of the transcriptomic data and identified a downregulation of the “epithelial-mesenchymal-transition” signature, which is known to overlap with ECM organization changes^22^, and of inflammatory pathways including “TNFα Signaling via NFκB” and “Inflammatory response” in LYN^KO^ fibroblasts (Fig. 3f, Supplementary Fig. 2j).

Together, LYN^KO^ HS-5 cells exhibit a uniformly altered profile on both protein and transcription levels which points to a transcriptional reprogramming function of LYN in BMSC. This rewiring of fibroblasts towards a leukemia-supportive phenotype seems to involve a profound remodeling of the ECM and of cytokine production.

To further validate these observations in vivo, primary murine fibroblasts (CD45^−^ CD31^−^ CD71^−^ CD29^+^ CD54^+^) from spleens of *Lyn*^*wt/wt*^ and *Lyn*^*−/−*^ mice were isolated and analyzed by bulk RNA-sequencing (Supplementary Fig. 2k). The sorting strategy successfully depleted immune-related genes and enriched stromal genes (Supplementary Fig. 2l, m). GO-Term enrichment analysis demonstrated a prominent deregulation of ECM-related molecular functions in murine splenic fibroblasts upon LYN deficiency (Supplementary Fig. 2n), mirroring our observation in HS-5 cells. However, splenic fibroblasts occurred at low abundance, and the analyzed cell populations still contained residual immune cells that might partially account for the changes in DEGs and deregulated pathways.

### LYN deletion disturbed the CAF-like phenotype in bone marrow stromal cells and primary CAF

Stromal cells in the malignant microenvironment acquire an adapted secretion of soluble factors and remodeling of the ECM, through which they influence cancer cell biology and disease progression^7^, altogether summarized as a cancer-associated fibroblast (CAF) phenotype. Similarly, stromal cells in the CLL-TME acquire a CAF-like phenotype contributing to leukemia progression^4,6^. As LYN^KO^ in stromal cells particularly affected cytokine- and ECM-related pathways and resulted in a reduced tumor support, we postulate that LYN expression in stromal cells may modulate CAF features.

**Fig. 4:**
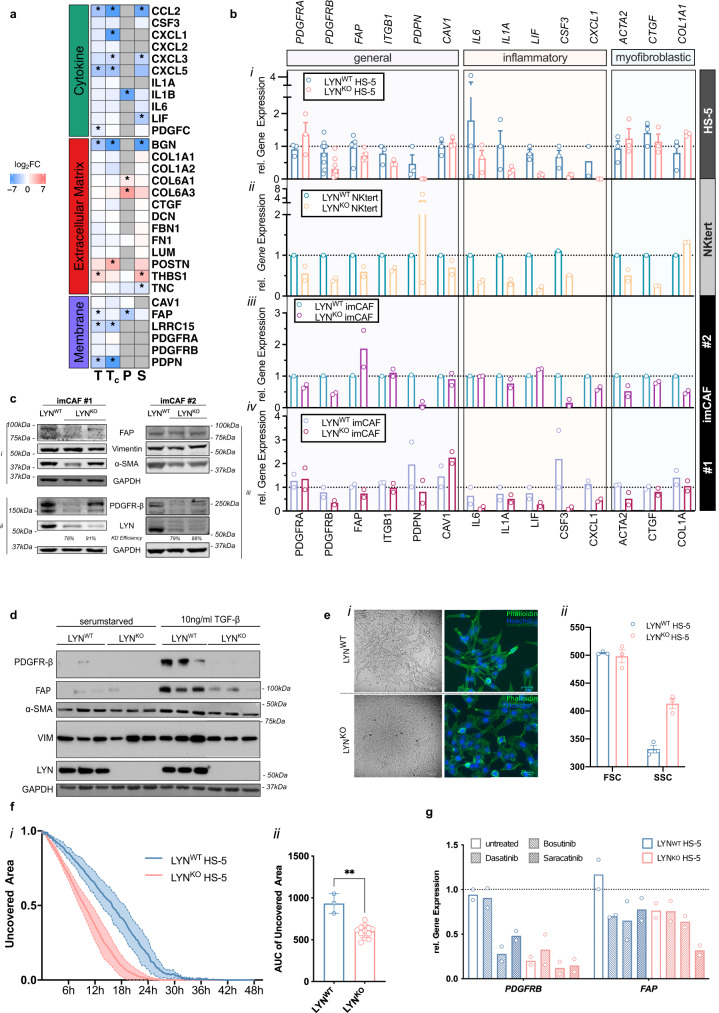
LYN deletion disturbed the CAF-like phenotype and associated polarization in stromal cells by diminishing inflammatory features and enhancing typical myofibroblastic functions. **a** Heatmap of a curated list of genes associated with CAF phenotype. Fold changes (log_2_-FC) from “omics”-layers are depicted. Red and blue color represent log_2_-FC of genes in LYN^WT^ and LYN^KO^ clones, respectively. Individual layers, where a gene was not detected, are gray. Genes were grouped by their cellular compartment and asterisks indicate “omics”-layers where genes were significantly differentially expressed (specified in Methods). **b** Transcriptional levels of CAF markers according to Biffi et al.^24^ in different LYN^WT^ and LYN^KO^ cells (*i*: HS-5, *ii*: NKtert, *iii/iv*: two imCAF lines) determined by qRT-PCR. (HS-5: 3 SCC per genotype; NKtert: 1 LYN^WT^ SCC vs. 2 LYN^KO^ SCC; imCAF#1: 2 polyclones per genotype, imCAF#2: 1 LYN^WT^ vs. 2 LYN^KO^ polyclones). **c** Immunoblot of LYN^WT^ or LYN^KO^ imCAF#1 and imCAF#2 for selected CAF markers. (i) and (ii) were prepared from the same lysates. **d** Immunoblot of LYN^WT^ or LYN^KO^ HS-5 cells (3 clones per genotype, untreated or stimulated with 10 ng/ml TGFβ for 24 h) for selected CAF markers. **e** (i) Bright field (left) and immunofluorescence microscopy (right) for phalloidin and Hoechst showing morphology of LYN^KO^ cells under normal culture conditions. (ii) Quantification of FSC and SSC of HS-5 LYN^WT^ and LYN^KO^ (mean ± SEM, 3 clones per genotype). **f** Migration of HS-5 cells in a scratch assay. The scratch area relative to the maximal value was measured every 20 min by live cell microscopy for HS-5 LYN^WT^ (1 clone) and LYN^KO^ (3 clones) in independent triplicates (mean ± SD) (i). Area Under the Curve (mean ± SD; Mann-Whitney test *p* = 0.0044) (ii). **g** LYN^WT^ and LYN^KO^ HS-5 cells were treated for 24 h with kinase inhibitors (1 µM dasatinib, 5 µM bosutinib, 5 µM saracatinib), mRNA levels of *PDGFRB* and *FAP* were assessed by qRT-PCR (2 SCC per genotype). In all qRT-PCR gene expression was normalized to *PPIA*. Depicted are means (± SEM where applicable) from the indicated number of clones per genotype. See also Fig. S3. Source data are provided as a Source Data file.

GSEA enrichment of experimentally defined genes upregulated in CAF from bone marrow derived mesenchymal stem cells published before^23^ indicated a significant deregulation of those in LYN^KO^ stroma cells (Supplementary Fig. 3a). Correspondingly, a manually curated set of “CAF related markers” identified numerous markers that were significantly altered in HS-5 LYN^KO^ cells (Fig. 4a), corroborating a role of LYN in the CAF differentiation.

To further validate changes in the CAF signature upon LYN deletion, we examined an array of typical CAF markers as previously defined in pancreatic cancer^24^ in four cell lines. Besides the two BMSC lines HS-5 and NKtert, we established two immortalized CAF (imCAF) cell lines from two patient-derived pancreatic cancer specimens and generated two polyclonal, partial LYN^KO^ clones using CRISPR/Cas9 in each imCAF line (Supplementary Fig. 3b). Strikingly, mRNA expression of various general CAF markers was consistently reduced upon LYN knockout (Fig. 4b): *PDGFRB* was clearly downregulated in all cell lines, and *FAP* and *ITGB1* mRNA levels showed a trend towards reduced expression. However, the levels of *PDGFRA* and *CAV1* were altered inconsistently. *PDPN* expression was completely absent in LYN^KO^ HS-5 and imCAF#2 cells, but exhibited variable levels in different NKtert and imCAF#1 clones. At the protein level, LYN expression was reduced by 78–91% in LYN^KO^ imCAF clones, which resulted in visible reduction of PDGFRβ and αSMA (Fig. 4c).

### LYN deficiency altered CAF-associated polarization by diminishing inflammatory features and enhancing typical myofibroblastic functions

CAF display very heterogenous polarization states and can be segregated into two most abundant subtypes with distinct phenotypes and functions: inflammatory (iCAF) and myofibroblastic (myCAF), with iCAF being largely responsible for cytokine secretion and myCAF for ECM remodeling^8,9^. We also assessed mRNA expression of markers associated with these subtypes^24^ and observed a consistent reduction in various inflammatory molecules, including *IL6*, *IL1A*, *LIF*, *CSF3* and *CXCL1* (Fig. 4b), strongly corroborating the described perturbation of cytokine-related pathways in HS-5 cells (Fig. 3c, d, f) and further implying an inhibition of inflammatory iCAF promoting pathways in the LYN^KO^ cells.

The transcriptional differences in myofibroblastic markers such as *ACTA2*, *CTGF* and *COL1A1* were less consistent than iCAF markers across the four cell lines (Fig. 4b). PDAC-derived CAF featured a myofibroblastic polarization with high αSMA expression, which was apparently decreased in LYN-deficient imCAF lines (Fig. 4c). Stimulation of LYN^KO^ HS-5 cells with the major myofibroblast promoting cytokine TGFβ completely failed to induce PDGFRβ and FAP expression in comparison to LYN^WT^ cells (Fig. 4d). Moreover, LYN^KO^ cells exhibited a more bulbous, rounded morphology distinct from the classical elongated, spindle-shaped LYN^WT^ cells (Fig. 4e) accompanied by an increase in flow-cytometric side-scatter which implies further changes of the intracellular structure in LYN^KO^ cells. Also, migration in a wound healing scratch assay was significantly enhanced in LYN^KO^ HS-5 cells (Fig. 4f). Altogether, this implies that LYN deficiency also impacts on the myofibroblast-related phenotype and function of stromal cells, closely related to the changes in ECM remodeling described in the multi-omics analysis.

Treatment of LYN^WT^ HS-5 cells with the different LYN/SFK inhibitors dasatinib, bosutinib and saracatinib exerted similar transcriptional changes as genetic LYN knockout: All inhibitors impaired *FAP*; dasatinib and saracatinib also impaired *PDGFRB* expression (Fig. 4g). The effects induced by these inhibitors appeared to be mainly mediated by LYN because *PDGFRB* and *FAP* expression were not additionally compromised by dasatinib and bosutinib in LYN^KO^ cells (Fig. 4g); only saracatinib further reduced the already decreased mRNA levels of *FAP* in LYN^KO^ HS-5 cells.

Although FAP and PDGFRβ expression was strongly diminished in LYN^KO^ BMSC (Fig. 4a–d, Supplementary Fig. 3c) and both molecules were associated with the leukemia protective function of stromal cells^25–27^, both FAP- and PDGFRβ-knockdown (Supplementary Fig. 3d) did not impair stromal feeding capacity in CLL co-culture (Supplementary Fig. 3e, f). This demonstrates that FAP and PDGFRβ are not the main effectors of LYN function in BMSC, and the perturbated CAF-program might rather be of collective relevance.

Altogether, these results show that LYN^KO^ fibroblasts resemble a disturbed CAF-like phenotype in different cell lines, impacting on the inflammatory differentiation and associated cytokine secretion as well as on the ECM-related myofibroblastic differentiation. Treatment with available SFK inhibitors further indicated the relevance of LYN in this reprogramming, as effectiveness of reducing FAP and PDGFRβ expression by kinase inhibition was impaired in LYN^KO^ stromal cells.

### Diminished CLL support upon LYN deletion is not related to changes in cytokine secretion

**Fig. 5:**
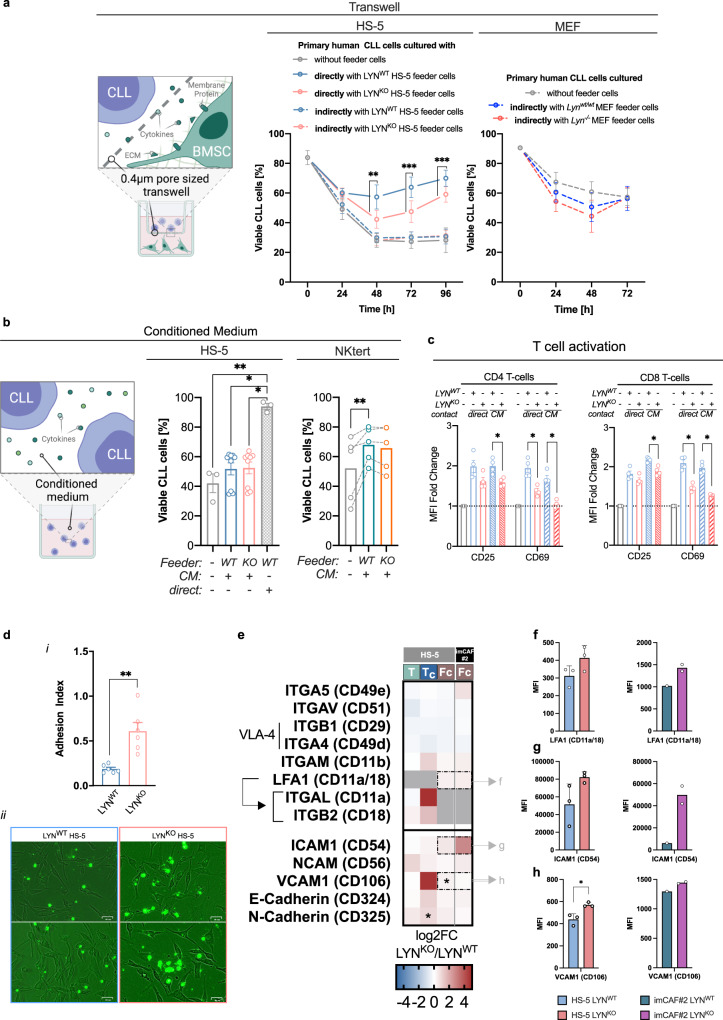
Diminished CLL support upon LYN deletion is not related to changes in cytokine secretion. **a** CLL cells (*n* = 5) were cultured with HS-5 (1 clone per genotype) (*middle*) or MEF (CLL: *n* = 4; MEF: 1 clone per genotype) (*right*) in direct contact or separated by a 0.4 µm pore-sized transwell membrane. (mean ± SEM; Uncorrected Fisher’s LSD test for LYN^WT^ vs. LYN^KO^: 48 h direct *p* = 0.0028; 72 h direct *p* = 0.0004; 96 h direct *p* = 0.0003). **b** CLL (*n* = 3) were cultured for 72 h in HS-5 cell conditioned medium (3 clones per genotype) (*middle*):, without feeder cells vs. direct contact to LYN^WT^ *p* = 0.0032, with CM from LYN^WT^ vs. direct contact to LYN^WT^ *p* = 0.0156, with CM from LYN^KO^ vs. direct contact to LYN^WT^ *p* = 0.0201) or 48 h in NKtert cell conditioned medium (*right*) (CLL: *n* = 5, NKtert: 1 clone per genotype, without feeder cells vs. CM from LYN^WT^ *p* = 0.0044), (mean ± SEM; two-sided Uncorrected Dunn’s test). **c**, Healthy PBMC (*n* = 4) were cultured for 72 h alone, in direct contact or with conditioned medium from LYN^WT^/LYN^KO^ HS-5 cells. MFI of activation markers on CD3^+^CD4^+^ or CD3^+^CD8^+^ cells were normalized to cells cultured alone (mean ± SEM; two-sided Mann-Whitney test against LYN^WT^ conditions: for all tests *p* = 0.028571). **d** CFSE-labeled CLL cells (*n* = 2) were co-cultured with LYN^WT^ or LYN^KO^ HS-5 cells for 48 h, images were taken after rigorous washing (*i*). Adhesion Index depicts CFSE^+^ CD45^+^ CLL cells per CFSE^-^ HS-5 cells (ii) (3 clones per genotype, mean ± SEM, two-sided Mann-Whitney test *p* = 0.0022). **e** Heatmap depicting expression fold change from HS-5 T and T_c_ data, and flow cytometry (Fc) of HS-5 and imCAF#2 cells. Color depicts log_2_-FC (LYN^KO^/LYN^WT^) of gene expression/MFI respectively, asterisks indicate statistical significance specified for each dataset). **f–h** MFI for (**f**) LFA1, (**g**) ICAM1 and (**h**) VCAM1 in HS-5 (*left*, 3 clones per genotype) and imCAF#2 *(right*, 1 LYN^WT^ clone vs. 2 LYN^KO^ clones) cells (mean ± SD, two-sided Welch’s t-test *p* = 0.0308). Illustration was created with BioRender.com. Source data are provided as a Source Data file.

Despite the profound changes in secreted cytokines of LYN^KO^ cells, the reduction of these soluble factors was insufficient to explain the altered feeder effect of LYN^KO^ cells on leukemic cells, particularly because direct contact is needed for CLL support^28^: Indirect co-culture separating feeder and leukemic cells via transwell membranes in HS-5 and MEF was incapable to increase CLL viability (Fig. 5a). Although HS-5 or NKtert conditioned medium could slightly enhance leukemic viability, this effect was negligible compared to direct co-culture and independent of stromal LYN-expression (Fig. 5b), indicating that stromal LYN supports CLL viability upon direct contact.

To investigate if the perturbated cytokine secretion upon LYN deficiency had other functional relevance because it has been increasingly acknowledged that CAF secretome can strongly modulate immunity via interaction with immune cells^11,29^, we assessed stromal LYN effect on T-cell activation. Culturing PBMC directly on HS-5 stroma cells or indirectly with HS-5 conditioned medium induced an increase in surface expression of CD25 and CD69 activation markers on CD4 and CD8 T cells, whereas this activation was significantly reduced upon culture with LYN^KO^ HS-5 cells (Fig. 5c). This suggests a potential impact of stromal LYN on T-cell-mediated immune response towards leukemia, driven by changes in cytokine secretion.

Despite the dependency of leukemic feeding capacity on direct contact, the reduced leukemic survival observed on LYN^KO^ cells was not caused by impaired adhesion because CLL cells could adhere 3-fold better to LYN^KO^ than to LYN^WT^ HS-5 cells after co-culture (Fig. 5d). The increased adhesion may relate to the elevated transcript and protein levels of several adhesion molecules such as LFA1, ICAM1 and VCAM1 on LYN^KO^ cells (Fig. 5e–h). Although adhesion of CLL cells to stroma via VLA4-VCAM1 and LFA1-ICAM1 signaling axes can facilitate leukemic cell survival^4,30,31^, their increased levels could not compensate for the loss of stromal LYN in this setting.

### LYN-dependent regulation of ECM proteins like Thrombospondin-1 (THBS1) in stromal cells governed the viability of CLL cells

**Fig. 6:**
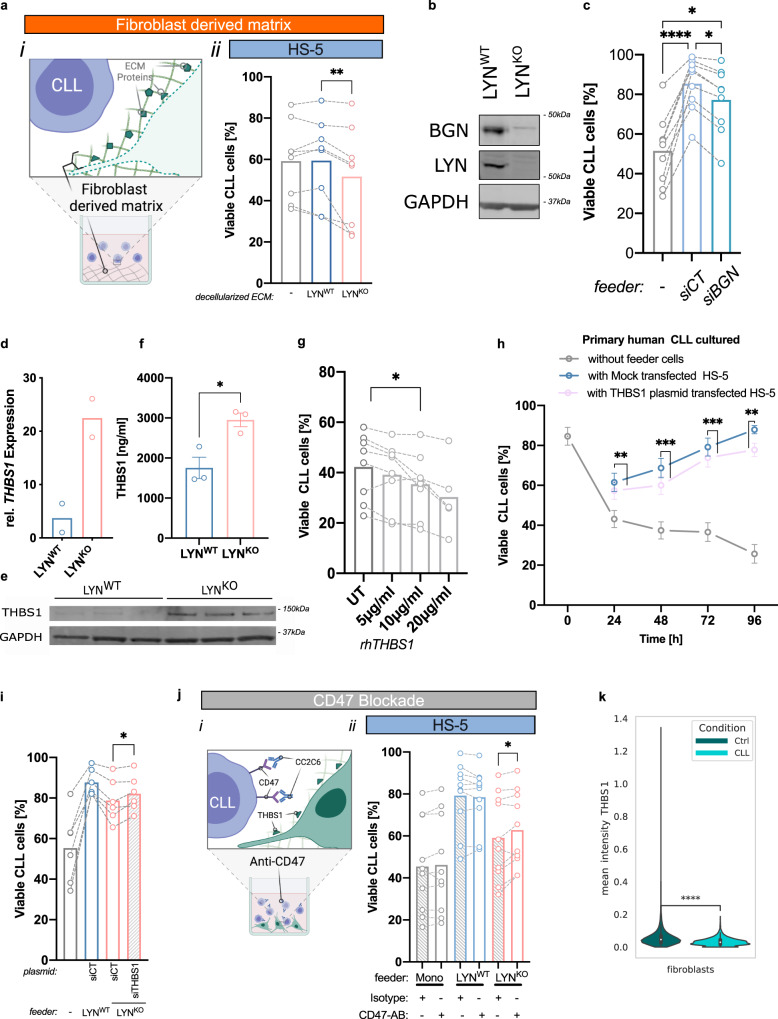
LYN-dependent regulation of the ECM proteins like Thrombospondin-1 (THBS1) in stromal cells supported the viability of CLL cells. **a** CLL cells (*n* = 8) were cultured on decellularized ECM from LYN^WT^ and LYN^KO^ HS-5 cells for 48 h. (i) illustration, (ii) CLL viability as Annexin V^−^/7AAD^−^ per CD45^+^ cells. (mean, two-sided Wilcoxon test, *p* = 0.0078). **b** Immunoblot of BGN in LYN^WT^ and LYN^KO^ HS-5 cells (1 sample each). **c** HS-5 cells were transfected with si*BGN* or control *siRNA* (15 nM), CLL (*n* = 9) were co-cultured for 96 h (mean, two-sided Uncorrected Dunn’s test: without feeder vs. siCT *p* < 0.0001; without feeder vs. siBGN *p* = 0.0339; siCT vs. siBGN *p* = 0.0339). **d–f** THBS1 expression in HS-5 cells. **d** qRT-PCR analysis normalized to *PPIA*. **e** Immunoblot for THBS1. **f** Soluble THBS1 measured by ELISA (**d**: 2 clones per genotype, **e**, **f**: 3 clones per genotype; mean ± SEM; unpaired two-sided t-test: *p* = 0256). **g** CLL cells (*n* = 8) were cultured with recombinant human THBS1 and viability was assessed after 24 h (mean, two-sided Wilcoxon test compared to untreated, *p* = 0.0391). **h** THBS1-overexpressing and Mock HS-5 cells were cultured with CLL cells (*n* = 15, 1 clone per condition), (mean ± SEM; two-sided Wilcoxon test for Mock- vs. THBS1-overexpressing: 24 h *p* = 0.005371, 48 h *p* = 0.000366, 72 h *p* = 0.000427, 96 h *p* = 0.003906). **i** CLL cells (*n* = 6) were co-cultured with THBS1 knockdown-LYN^KO^ or control HS-5 cells for 72 h (mean, two-sided Wilcoxon test *p* = 0.0312). **j** CLL cells (*n* = 10) were co-cultured with antibody-pretreated HS-5 cells for 48 h. (i) illustration, (ii) CLL cell viability was quantified by flow cytometry (mean, two-sided Wilcoxon test *p* = 0.0469). **k** Violin plot for mean THBS1 expression per segmented single cell in Imaging Mass Cytometry (Fig. 1d). HC-LN: *n* = 9 (6466 fibroblasts), CLL-LN: *n* = 12 (7543 fibroblasts). Boxplots represent median with the interquartile range, whiskers indicate adjacent values, violin represents kernel density estimation, independent two-sided t-tests (*p* = 1.522e−257). See also Fig. S4. Illustration was created with BioRender.com. Source data are provided as a Source Data file.

As ECM generally shapes lymphomagenesis^12,32^ and can promote viability and chemoresistance of CLL via molecules such as CD44^33^ or integrins^4^, we hypothesized that LYN effects were mediated by CLL contact to the ECM. Indeed, CLL cells cultured on decellularized fibroblast derived matrices (FDM) from LYN^KO^ HS-5 cells showed a significantly reduced viability compared to LYN^WT^ FDM co-culture (Fig. 6a). Notably, while LYN^WT^ FDM did not clearly influence CLL viability when compared to mono-culture, LYN^KO^ FDM surprisingly reduced CLL viability to lower levels than CLL mono-culture, suggesting that LYN^KO^ ECM may eventually exhibit leukemia-suppressive functions. In conclusion, the changes in the ECM seem to be highly relevant in attenuating CLL cell viability upon direct stromal contact.

To dissect the functional relevance of additional, specific ECM components, we investigated the ECM protein Biglycan (BGN), which was found almost absent on protein and transcript levels in LYN^KO^ HS-5 cells in multi-omics analyses (Fig. 3b, Supplementary Fig. 2d, e, g). Immunoblotting validated the strongly reduced protein expression upon LYN deficiency (Fig. 6b) and importantly, siRNA-mediated knockdown of BGN in HS-5 cells resulted in a significant reduction of CLL cell survival in co-culture experiments (Fig. 6c). This suggested a leukemia-supportive function of BGN that is lost after ECM remodeling in LYN^KO^ stromal cells.

However, loss of leukemia-supportive BGN alone does not explain the leukemia-restrictive effect of LYN^KO^ FDM. Tumor-suppressive functions have been described for several ECM proteins in solid cancers^8^ and some hematological malignancies^12–14^. In CLL, the matrix glycoprotein Thrombospondin-1 (THBS1), a versatile player of the microenvironment, is capable of inducing caspase independent apoptosis of leukemic cells via CD47 ligation^34–36^, and increased THBS1 levels are associated with better survival of CLL patients^13^. Interestingly, increased levels of the CLL-restrictive matrix protein THBS1 were detected in the transcriptome and secretome of LYN^KO^ HS-5 cells (Fig. 3b, Supplementary Fig. 2d, e), potentially explaining this leukemia-restrictive ECM in LYN^KO^ BMSC. Validating the multi-omics results, mRNA expression of THBS1 was increased 6-fold in LYN^KO^ HS-5 and 50-fold in NKtert cells, respectively (Fig. 6d, Supplementary Fig. 4a). The THBS1 protein was strongly enriched in LYN^KO^ HS-5 cells and culture media compared to LYN^KO^ counterparts (Fig. 6e, f). In imCAF cells, THBS1 protein was also overexpressed in LYN^KO^ clones, but showed variable mRNA levels (Supplementary Fig. 4b, c).

In agreement with a previous report^35^, recombinant human THBS1 reduced CLL cell viability in a time and dose dependent manner after 6 h and 24 h in vitro (Fig. 6g, Supplementary Fig. 4d). Additionally, the co-culture of CLL cells with THBS1-overexpressing HS-5 cells similarly decreased CLL viability compared to controls (Fig. 6h, Supplementary Fig. 4e) as early as 24 h after co-culture. THBS1 overexpression had no effect on feeder cell proliferation or viability (Supplementary Fig. 4f). Reversely, siRNA-mediated knockdown of the overexpressed THBS1 in LYN^KO^ HS-5 cells (Supplementary Fig. 4g) increased the feeding capacity of LYN^KO^ cells in co-culture, thereby partially reversing the effects of the LYN knockout (Fig. 6i) without affecting feeder cell proliferation (Supplementary Fig. 4h). Moreover, pretreatment of CLL cells with a blocking anti-CD47 antibody induced a significant increase of CLL cell viability in co-culture with LYN^KO^ HS-5 but had no effect on viability in LYN^WT^ co-culture (Fig. 6j), mechanistically confirming that THBS1 induced CLL cell death via CD47 ligation. Notably, the used anti-CD47 antibody acted as a blocking antibody, and did not impair CLL cell viability (Supplementary Fig. 4i). The distinctive efficacy of CLL-specific CD47 blockade in co-culture between LYN^WT^ and LYN^KO^ BMSC strongly supports the notion that LYN regulates THBS1 expression in stromal fibroblasts and acts—at least in part—via CD47 ligation on CLL cells. In accordance with the inhibitory effect of LYN on THBS1 expression in cell lines, we observed significantly lower levels of fibroblastic THBS1 in CLL-LN compared to HC-LN in imaging mass cytometry analysis (Fig. 6k, Supplementary Fig. 4j).

Collectively, LYN^KO^ stromal cells displayed a leukemia-restricting ECM composition, exemplified by the significant modulation of THBS1 and BGN. As expected, modulation of either THBS or BGN alone could not fully recapitulate stromal LYN^KO^ effect on CLL cell survival, suggesting that LYN kinase orchestrates additional regulators.

### A perturbated inflammatory signaling and a reduced cJUN expression disinhibit THBS1 expression and suppress leukemia support in LYN^KO^ BMSC

**Fig. 7:**
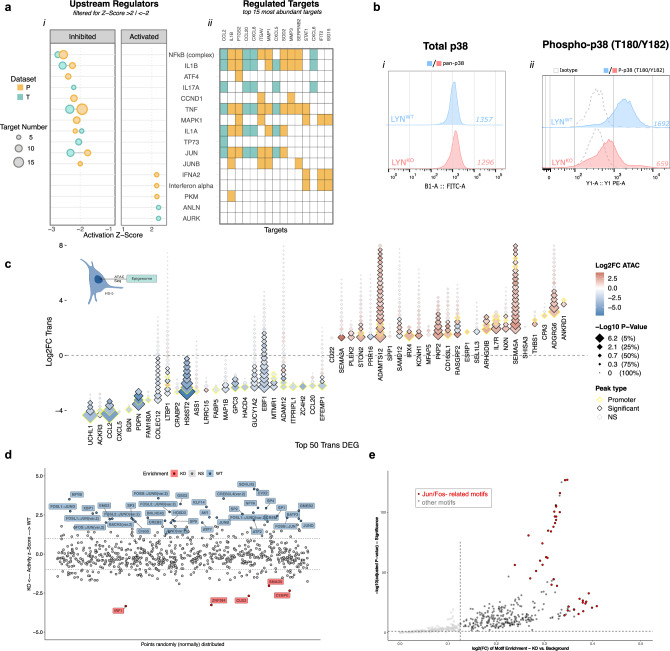
Inflammatory signaling involving p38 phosphorylation and c-JUN transcriptional activity was perturbated upon LYN deficiency. **a** Ingenuity Pathway Analysis (IPA) of Transcriptome (T) and Proteome (P). (i) Predicted upstream regulators with an absolute mean activation Z-score >2 are depicted. Individual T and P “omics”-layers are colored respectively and the circle size determines the number of associated target genes. (ii) Heatmap of the 15 most abundant target genes of the selected upstream regulators, showing gene-regulator associations. **b** Histogram of flow-cytometric assessment of total (i) and Phospho-T180/Y182 (ii) p38 MAPK expression in unstimulated HS-5 LYN^WT^ and LYN^KO^ cells (1 representative clone per genotype of 3 clones). Dashed curves represent isotype controls, numbers indicate uncorrected MFI. **c** Diamond plot illustrating homogenous relation between Epigenomic and Transcriptomic changes in HS-5 cells. For the top 50 DEGs (25 genes each with biggest/smallest log_2_-FC) from HS-5 Transcriptome (T), each diamond represents one related accessible-interval measured in HS-5 ATAC-sequencing data. Y-Axis position of the lowest diamond illustrates log_2_-FC of the representative gene in HS-5 Transcriptome. Color of each diamond represents log_2_-FC and size −log_10_ *P* value of the respective interval in HS-5 ATAC-Seq data (analysis specified in Methods). Black margin highlights differentially accessible (DA) intervals, yellow margins DA-promoter intervals. Graphical illustration was created with BioRender.com. **d** Transcription factor footprinting analysis of HS-5 Epigenome. Activity plot showing the distribution of z-transformed activity scores of the underlying motifs (y-axis). On the x-axis the points are distributed randomly. Motifs with differential footprinting (*p* value ≤ 0.05 and |z-Score | ≥ 1, analysis specified in Methods) are highlighted in red (enriched in LYN^KO^) or in blue (enriched in LYN^WT^). **e**, One-sided Volcano plot of HS-5 Epigenome motif enrichment analysis. X-Axis shows the ratio of foreground (in DA-intervals) vs. background (in all intervals) motif frequency as log_2_-FC, y-Axis shows the respective adjusted *p* value (analysis specified in Methods). JUN/FOS-related motifs are highlighted in red. See also Fig. S5. Source data are provided as a Source Data file.

To elucidate the regulatory mechanism underlying LYN-induced effects and predict the involved upstream regulators, Ingenuity Pathway Analysis (IPA) was performed (Fig. 7a). Corresponding to changes in iCAF signature (Fig. 4b) and reduced inflammatory signatures in GSEA (Fig. 3f) we found a striking reduction of an inflammatory regulative network, including the NFκB complex, TNF, IL1 and JUN in LYN^KO^ cells (Fig. 7ai), which was mainly driven by differential expression of cytokines such as CCL2, IL1, CXCL8, CXCL5 and CCL20, and of ECM components like ITGAV, MMP1 and MMP3 (Fig. 7aii). Interestingly, IPA revealed that inflammatory pathways were affected differentially by LYN depletion, highlighting an upregulated Interferon alpha (IFNA2) signaling in LYN^KO^ cells. This reflected the enrichment of Interferon alpha and Interferon gamma signatures in GSEA analysis (Fig. 3f, Supplementary Fig. 2j), upregulation of STAT1 and the Interferon response genes IFIT1 and IFIT2 on mRNA and protein level (Fig. 3b, Supplementary Fig. 2c) and an increased phosphorylation of STAT1-Y701 in pYome (Supplementary Fig. 2f).

The identified upstream regulators implied deregulation of MAPK signaling pathway, integrating extracellular ligands such as IL1, IL17 and TNF, MAPK1 (ERK2) kinase and the transcriptional factors JUN and JUNB. X2K analysis on DEG/Ps from P and T, which performs transcription factor enrichment and subsequent upstream kinase enrichment analysis^37^, similarly indicated deregulation of kinases related to MAPK signaling, in particular MAPK14 (p38 Kinase) (Supplementary Fig. 5a). Moreover, a reduced phosphorylation at the activating MAPK14-Y182 site was amongst the strongest alterations detected in pYome data (Supplementary Fig. 2f) and was validated by flow cytometry (Fig. 7b), strongly endorsing changes in MAPK signaling induced by the LYN knockout in fibroblasts.

To further dissect the downstream transcriptional regulation, ATAC-sequencing of LYN^WT^ and LYN^KO^ HS-5 cells demonstrated profound changes in accessible chromatin with a generally more open state in LYN^WT^ cells (Supplementary Fig. 5b, c). Genes with the most reduced transcripts in LYN^KO^ cells (*CCL2, BGN, PDPN*) also showed significantly reduced chromatin accessibility in multiple gene intervals, including significant deregulation in promoter accessibility (Fig. 7c). Likewise, genes with the highest increase of transcripts showed increased chromatin accessibility in LYN^KO^ cells (*ADGRG6, THBS1, IL7R*), verifying that LYN^KO^-mediated stromal reprogramming occurs largely at the transcriptional level. Footprinting analysis, which characterizes the underlying changes in transcription factor (TF) activity, identified several TF with higher DNA binding in LYN^WT^ cells like SP and CREB. In LYN^KO^ cells, AP-1 complex dimers showed a particularly reduced activity compared to LYN^WT^ cells (Fig. 7d), which could be recapitulated in TF-motif enrichment of differentially accessible intervals (Fig. 7e). These data supported the IPA upstream regulator predictions pointing towards deregulated MAPK signaling that affected AP-1/JUN activity. On the other hand, only a few pathways were more active in LYN^KO^ cells, with IRF1 pathway showing the most prominent increase, which parallels the increased expression of interferon signatures observed in the LYN^KO^ cells in multi-omics analysis.

**Fig. 8:**
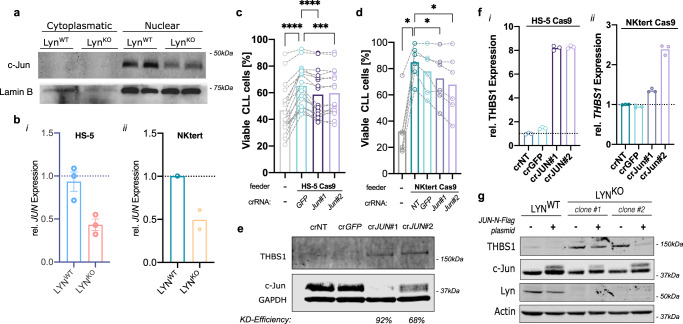
Reduced c-JUN expression disinhibited THBS1 expression and suppressed leukemia support in BMSC. **a** Immunoblot of total c-JUN protein expression in cytoplasmatic and nuclear fractions of LYN^WT^ and LYN^KO^ HS-5 cells (2 clones per genotype). **b** Transcriptional expression of *JUN* in (i) HS-5 (3 clones per genotype), (ii) and NKtert (LYN^WT^ 1 clone, LYN^KO^ 2 clones). Expression was measured by qRT-PCR and normalized to *PPIA* housekeeping gene expression (mean ± SEM). **c** HS-5 Cas9 cells were transfected with different crRNA sequences targeting *GFP* or *JUN* 5 days before they were co-cultured with primary human CLL cells for additional 48 h. CLL cell viability in co-culture was quantified by flow cytometry as Annexin V^-^/DAPI^-^ of all CD45^+^ cells (CLL *n* = 17; mean; Wilcoxon rank test compared to crGFP: without feeder and crJUN#1 *p* < 0.0001; crJUN#2 *p* = 0.0004). **d** NKtert Cas9 cells were transfected with different crRNA sequences targeting *GFP*, *JUN* or non-targeting (NT) 3 days before they were co-cultured with primary human CLL cells for additional 72 h. CLL cell viability in co-culture was quantified by flow cytometry as Annexin V^−^/DAPI^−^ of all CD45^+^ cells (CLL *n* = 6; mean; two-sided Wilcoxon rank test compared to crNT: in all tests *p* = 0.0312). **e** Immunoblot of JUN^KO^ (2 samples with different gRNAs) and control HS-5 (2 independent samples) Cas9 cells showing THBS1 and c-JUN protein expression. JUN knockout-efficiency was calculated to crNT control. **f** qRT-PCR analysis of relative *THBS1* mRNA expression normalized to *PPIA* housekeeper gene expression of HS-5 Cas9 cells (i) and NKtert Cas9 cells (ii) following CRISPR-mediated JUN knockout (mean, 3 technical replicates). **g** Immunoblot of LYN^WT^ (one SCC) and LYN^KO^ (2 SCCs) HS-5 cells showing THBS1 and c-JUN protein expression 72 h after transduction with cJUN-N-Flag expressing plasmid or Mock control. See also Fig. S5. Source data are provided as a Source Data file.

Accordingly, reduced protein levels of nuclear c-JUN by approximately 50% were confirmed in HS-5 LYN^KO^ cells (Fig. 8a, Supplementary Fig. 5d), and both BMSC lines HS-5 and NKtert (Fig. 8b) as well as imCAF#2 (Supplementary Fig. 5e) exhibited diminished *JUN*-mRNA levels upon LYN deficiency. We explored the functional relevance of JUN in stromal cells by generating polyclonal, partial *JUN* knockout clones in HS-5 and NKtert stroma cells using two different gRNAs (Supplementary Fig. 5f, g). Upon co-culture with primary human CLL cells, both gRNA generated JUN^KO^ clones in the BMSC cell lines showed a reduced support of CLL growth compared to JUN^WT^ control cells (Fig. 8c, d). This illustrated that the leukemia-supportive LYN effects in BMSC were at least partially mediated by c-JUN.

Moreover, THBS1 transcription can be regulated by transcriptional factors downstream of the MAPK pathway such as c-JUN^38,39^. JUN^KO^ cells demonstrated an enhanced THBS1 expression on protein level (Fig. 8e) and a strong increase in mRNA level (Fig. 8f). To validate the regulatory function of c-JUN on THBS1 expression, c-JUN expression was reinduced in LYN^KO^ HS-5 cells, which reverted the increased THBS1 expression in LYN^KO^ cells (Fig. 8g, Supplementary Fig. 5h). This strongly suggested that overexpression of THBS1 upon LYN deficiency was mediated via a reduced expression of inhibitory c-JUN. Collectively, these results strongly indicate that a perturbation of inflammatory signaling contributes to the reprogramming of stromal cell following LYN deficiency and mechanistically, a decreased MAPK activity and diminished c-JUN expression is primarily responsible for the induction of THBS1 expression in LYN^KO^ stromal cells.

## Discussion

**Fig. 9:**
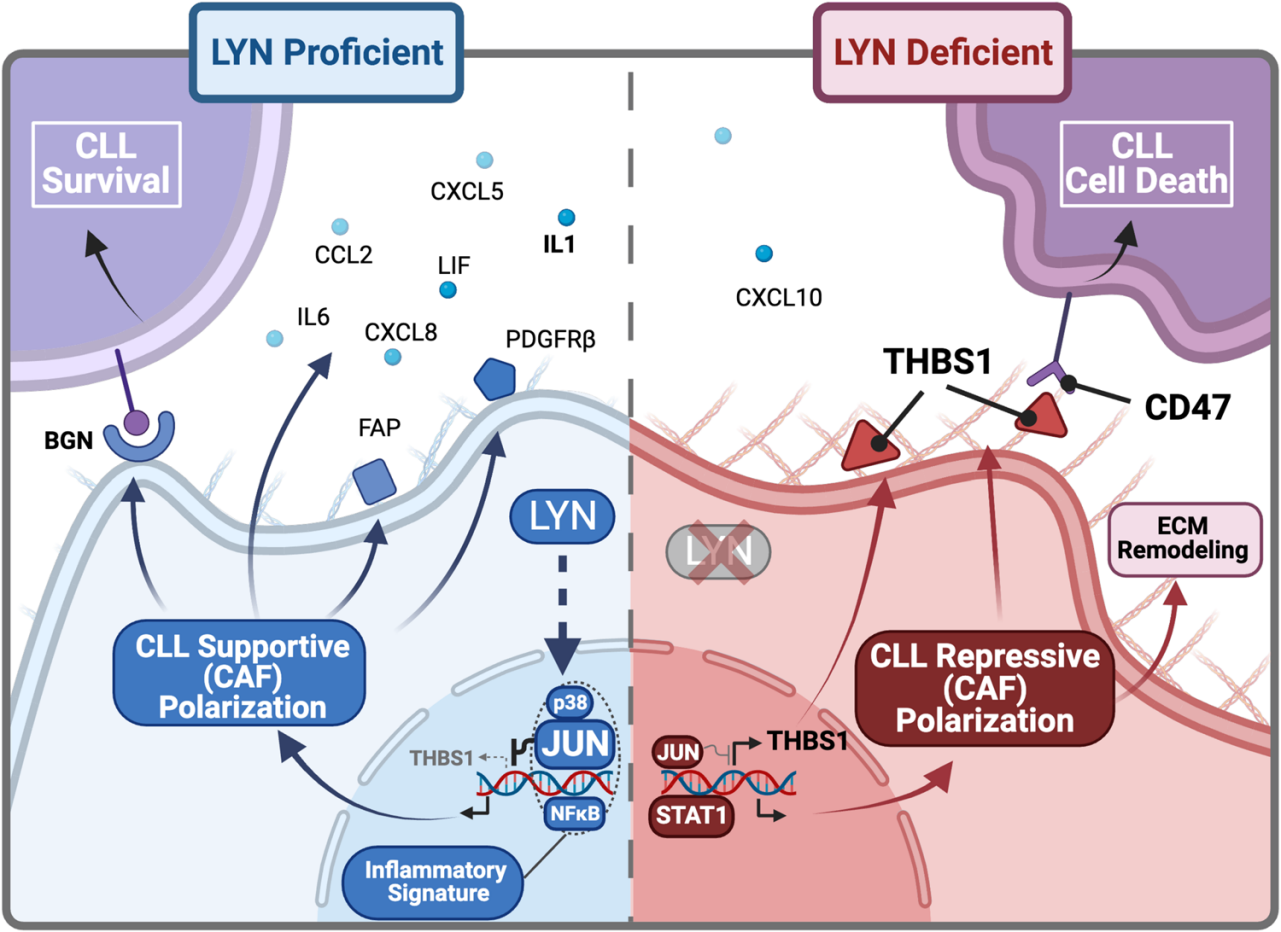
Graphical summary. Summary of LYN governing stromal polarization and regulating CLL support. *Left*: LYN induces transcriptional programming of BMSC via inflammatory regulators (p38, JUN, NFκΒ), promoting an iCAF-like polarization and suppressing THBS1 transcription. Subsequently, related cytokines (IL1, IL6, CCL2, CXCL5, CXCL8, LIF) and surface proteins (FAP, PDGFRβ) are highly expressed and CLL survival is fostered via direct cell contact, exemplarily via the ECM protein BGN. *Right*: In the absence of LYN, BMSC polarization is transcriptionally reprogrammed (reduced JUN, increased STAT1 expression), resulting in a reduced inflammatory cytokine secretion and diminished expression of iCAF markers. The ECM is remodeled with increased collagen production, and THBS1 expression is disinhibited by the reduced JUN expression. Direct contact to the remodeled ECM restrains CLL survival via enhanced THBS1-CD47 axis. Illustration was created with BioRender.com.

In this study, we report that the presence of LYN kinase in the stromal microenvironment supports leukemic cell survival by shaping the CAF-like polarization of stromal cells and maintaining a tumor supportive ECM composition, providing strong evidence for an eminent function of LYN in the tumor microenvironment. In an in vivo model, expansion of leukemia was delayed, and survival of mice significantly increased by selective ablation of LYN kinase within the non-hematopoietic microenvironment. In line with this, deletion of LYN in stromal cells in vitro consistently decreased the capacity of stromal cells to support leukemic cell survival, which was dependent on the direct contact of these cell types. Moreover, we showed that the loss of LYN attenuated inflammatory signatures including c-JUN expression, and induced profound alterations of cytokines and ECM proteins, particularly the upregulation of THBS1, which induces CLL cell death via CD47 ligation (Fig. 9). These findings were strongly supported by the observation that LYN expression was significantly enhanced in lymph node fibroblasts of CLL patients compared to healthy controls, accompanied by lower levels of fibroblastic THBS1 in CLL than healthy controls.

This study extends our previous observations regarding the regulatory role of LYN in the tumor microenvironment^2,20^. Previous studies in hematopoietic cells have shown that LYN is critical for regulating cellular homeostasis in a cell type- and context dependent manner^40^. LYN regulates the equilibrium of the BCR response^41^, and maintains a balanced response of myeloid cells by regulation of the Toll-like-receptor/MyD88 signaling^42–45^ and NLRP3 inflammasome^46^. Global- as well as B cell- and dendritic cell-specific *Lyn-*knockout in mice promotes hyperinflammation^41,42,46,47^, with global *Lyn*^*−/−*^ mice having a milder phenotype than mice with LYN-defective hematopoietic cells. Our results corroborate the hypothesis that the absence of LYN in non-hematopoietic cells may restrain hematopoiesis-induced inflammation^42^, by demonstrating a consistent downregulation of inflammatory gene signatures and regulators in LYN^KO^ stromal cells. This would suggest that LYN exerts different functions in stromal cells than in hematopoietic cells: predominant activation of inflammatory responses in the former and attenuation of inflammatory responses in the latter, which cooperatively results in a balanced intercellular homeostasis in the physiological and leukemic microenvironment^48^.

To date, studies about LYN in non-hematopoietic cells mostly focused on intrinsic functions of LYN in malignant cells^40,49–51^. Our findings provide evidence that LYN is shaping the cellular polarization of different cell types to support the development of CLL and presumably of other malignancies. We and others previously showed that LYN-deletion shifts the polarization of monocytes and macrophages towards an M2 phenotype^20,52^. This parallels our observation here of an even more pronounced shift in cellular plasticity upon LYN-deletion in stromal cells, strongly suggesting a role of this kinase in regulating cellular differentiation.

Stromal cells adapt to the leukemic microenvironment by acquiring a CAF-like polarization^53^, exemplarily shown in murine MSC upon CLL co-culture^6^. CLL-derived extracellular vesicles also induced this CAF-like phenotype in stromal cells, mainly by promoting inflammatory signaling via AKT and ERK, but interestingly also included LYN phosphorylation^5^. It was shown that CLL-EV can increase LYN expression in fibroblasts^54^. Based on our integrative multi-omics analysis, we argue that LYN in fibroblasts orchestrates in general inflammatory signaling events and in particular the CAF-like polarization of stromal cells, inducing widespread changes in gene signatures, marker expressions and tumor-promoting functions.

Our data corroborate that modulating protein kinases, especially those regulating pro-inflammatory pathways like NFκB or MAPK-JUN, alters stromal cell functions that are critical for the contact-mediated support of leukemia growth. In CLL, protein kinases in the stroma such as PKCβ promote disease progression and influence therapy response^6^. Besides inducing metabolic changes, PKCβ activates NFκΒ and TFEB in stromal cells, thereby regulating proteins involved in CLL contact like VCAM1^30,55^. We similarly demonstrated that stromal LYN stimulated comparable inflammatory signaling pathways and sustained CLL survival in a contact-dependent manner, agreeing with previous studies that direct cell contact was required for the feeding of CLL cells^30,56–58^. Moreover, we observed an increased adhesion of LYN^KO^ BMSC to CLL cells without providing sufficient survival support, suggesting that not only the strength of the adhesion signals but also the presence of appropriate survival signals or absence of restrictive signals from stromal cells is needed to ensure CLL growth. Considering the elevated levels of THBS1 upon LYN deletion, increased adhesion might even reinforce THBS1-CD47 crosslinking to drive CLL cell death.

THBS1, a relevant ECM component in many cancers,^59^ was shown to induce apoptosis by ligation to CD47^34–36^. Therefore, THBS1 upregulation through LYN-deficiency in fibroblasts reduced CLL survival in co-culture. As we demonstrated that LYN had a broad range of effects on the phenotype of stromal fibroblasts, it was not surprising that THBS1 overexpression in BMSC did not fully recapitulate the effects of LYN-knockout on CLL viability. Also, blocking the THBS1/CD47 signaling axis did not fully restore the feeder capacity in LYN-defective fibroblasts. Instead, LYN is regulating a group of ECM proteins such as Thrombospondin-1 or Biglycan, all contributing to the cumulative effects observed in LYN-defective cells. The identified LYN-dependent function illustrates how kinases can affect the overall ECM composition and the balance of pro- and anti-leukemic molecules of the CLL microenvironment. The reported connections between LYN and myofibroblast/fibrosis formation in organ fibrosis models^60^, as well as regulation of EMT^50,51^ are in line with our observation of a diminished EMT-signature and reduced responsiveness of CAF markers to TGFβ stimulation in LYN-deficient cells and further reflect the potential of LYN kinase to regulate the ECM composition. Moreover, as deletion of LYN partially mimics the inhibition of TGFβ (reduced EMT signature and TGFβ target genes like *MMPs, VCAN, BGN* and *LRRC15*, increased IFN-signatures)^61,62^, it is tempting to speculate that stromal LYN might be involved in TGFβ signaling. This notion may be supported by the reduced activation of p38 kinase, a downstream component of the non-canonical TGFβ signaling^63^. Moreover, interactions of LYN with involved upstream kinases of the SMAD-independent TGFβ signaling such as TRAF6 and TAK1 have been reported in mast cells^64,65^.

ECM seems to play an important role in hematological malignancies. For example, a mesenchymal gene signature was identified as a marker for a favorable prognostic outcome, and CAF-derived ECM molecules were linked to lymphoma-suppressive functions^12,14,18^. Moreover, ECM-related proteins (Col1A, Integrinα-V, Versican, THBS1) were associated with tumor suppression and better outcome in CLL^13^. Therefore, fibroblastic stromal cells may also have anti-leukemic functions, as demonstrated here for THBS1, similar to some of the tumor-suppressive functions of myCAF in solid cancers^8^. This highlights that a fine-tuned regulation of stromal cells and the ECM is important for optimizing their leukemic feeder capacity. The significantly lower level of THBS1 in CLL-LN fibroblastic cells suggests that suppression of leukemia-inhibitory factors in the stroma may represent a mechanism to promote tumor progression.

Kinase inhibitors are now routinely used for treating hematological malignancies. The modulation of non-malignant bystander cells is not only an apparent off-target effect of these inhibitors, but also contributes importantly to the mechanisms of action of kinase inhibitors in CLL^2^. A growing body of evidence points to functional roles of several protein kinases including LYN to modulate the phenotype of CLL bystander cells^6,20,66^, shaping the CLL niche. In solid cancers, researchers currently start to exploit the therapeutic potential of kinase inhibitors that modulate CAF plasticity^26^. A potential strategy to make some progress in this direction might be antibodies targeting CD47, which are emerging for the treatment of hematologic malignancies^67,68^. Besides enhancing the phagocytosis of malignant cells by macrophages, some antibodies might induce CD47 signaling and subsequent cell death of leukemic cells, similarly to stromal THBS1. Our study calls for further investigations on the role of kinases and their effectors in the CLL stroma. The increased use of single-cell RNA sequencing as well as murine models with lineage-specific kinase modifications will be beneficial for an improved mechanistic understanding of these interactions and their clinical use.

Together the results reported here support the concept that LYN modulates the polarization of stromal fibroblasts and induces an inflammatory CAF phenotype, thus rewiring essential components of a supportive microenvironment niche for leukemia cells. LYN-defective fibroblasts acquire leukemia-suppressive properties, mediated—at least in part—by a remodeled matrix and the increased expression of THBS1, which induces CLL cell death.

## Methods

### CLL samples

Primary CLL cells isolated from the peripheral blood of CLL patients, formalin-fixed paraffin-embedded primary CLL lymph nodes and lymphoid tissues from healthy donors were collected at the University Hospital of Cologne after written and informed consent according to the Declaration of Helsinki and with Institutional Review Board approvals at the University of Cologne no. 11-319, no. 13-091 (BioMaSOTA), no. 19-1559 (Buffy Coats), no 21-1317 (SFB 1530), no. 19-1438, no. 19-1438_1, and no. 21-1472. PBMC isolation and CLL cell purification procedures were previous described in ref. ^20^. CLL cells were stored frozen at −150 °C and thawed freshly for every assay.

### Mouse experiments

In vivo studies were approved by the state authorities of North Rhine-Westphalia, Germany (Landesamt für Natur-, Umwelt- und Verbraucherschutz Nordrhein-Westfalen (LANUV), approval no. 84-02.04.2016.A058). Only mice of C57BL/6-J background were used and maintained in a specific and opportunistic pathogen free (SOPF) animal facility under in-house standard housing conditions.

#### Bone marrow transplantation (BMT)

BMT, adoptive transfer of murine CLL cells and all procedures for blood sampling were described before^20^. In brief, 8 to 10 weeks old *Lyn*^*−/−*^ recipient mice were whole-body irradiated in the rodent gamma irradiation device Biobeam GM 8000 (Gamma-Service Medical, Leipzig, Germany) with a total dose of 11 Gy in 2 fragments. Four hours after the last irradiation, 10^7^ T-cell-depleted bone marrow cells from *Lyn*^*wt/w*t^ or *Lyn*^*−/−*^ donor mice were injected intravenously into the irradiated hosts. Successful reconstitution of the immune system after 8 weeks was assessed by peripheral blood sampling and flow-cytometric analysis. Chimeric mice were subsequently injected intraperitoneally with 10^7^ lymphocytes containing >90% CLL cells from *TCL1*^*tg/wt*^ mice, and leukemia burden was monitored by flow cytometry of peripheral blood samples every 14 days (all antibodies are listed in Supplementary Table 1). Maximal CLL burden was defined in the ethics approval as leukocyte number/µl mouse blood >100,000 cells. This maximal burden was not exceeded in any of the mouse experiments.

#### Short-term in vivo homing assay

Splenic cells of moribund *TCL1*^*tg/wt*^ mice were labeled with 5 µM CFSE (Abcam) and 10^7^ cells were injected intravenously into 8–12-week-old *Lyn*^*wt/wt*^ or *Lyn*^*−/−*^ mice. Recipient animals were sacrificed 4 h later to harvest cells from spleens and bone marrow (femur and tibiae). Cavities of the bones were flushed with PBS to access bone marrow cells. Flow-through marrow cells and splenocytes were passed through 100 µm strainers and cells were washed, incubated with FC-blocking solution (Miltenyi), fixed and permeabilized by the IntraPrep kit (BD Pharmingen) according to the manufacturer’s instructions. Cells were stained with fluorochrome labeled antibodies (see Supplementary Table 1) and measured by flow cytometry. Migrated CLL cells were identified as CFSE^+^/TCL1^+^ and normalized to the total cell count.

### Cell culture

HS-5 bone marrow stroma cells (RRID:CVCL_3720)^69^ were cultured in RPMI (Gibco) supplemented with 10% fetal bovine serum (FBS) (Gibco) and 1% Penicillin/Streptomycin (P/S) (Gibco). For TGFβ stimulation, cell layers were washed and incubated with 10 ng/ml TGF-β (CellSignaling) in serum-free RPMI medium for 24 h. NKtert cells (RRID:CVCL_4667)^70^ were cultured in DMEM/F12 (Gibco) supplemented with 10% FBS, 10% human serum (Sigma), 1% P/S, 0.2 µg/ml β-Mercapto-Ethanol (Gibco) and 0.45 µg/ml Hydrocortisone (Sigma). HUVEC cells (RRID:CVCL_9Q53) were cultured in EGM-2 endothelial cell growth medium-2 (LONZA) supplemented with 1% P/S. Human CAFs were isolated and purified from human pancreatic cancer tissue by a modified outgrowth method as first described by Bachem et al^71^. Freshly resected pancreatic tumor tissues were cut into small pieces and cultured in DMEM/F12 (Gibco) supplemented with 10% FBS (Gibco) and 1% P/S (Gibco) in a 6-well plate pre-coated with Collagen type I. Fibroblasts were let to migrate out the tissue fragments for 1–2 weeks and then cells were harvested with trypsin-EDTA and transferred to T75 flasks or 10 cm petri dishes for further purification and expansion. CAFs were immortalized with Lenti-virus induced SV40 small + Large T^72^. MEF cells were isolated from 13-day old embryos of *Lyn*^*wt/wt*^ and *Lyn*^*−/−*^ mice as described before^73^ and cultured in DMEM supplemented with 10% FBS and 1% P/S. All cell cultures were maintained in a fully humidified atmosphere with 37 °C, 5% CO2.

#### Co-culture and viability measurement of CLL cells

Before co-culture, 2.5 × 10^4^ (HS-5 and NKtert), 5 × 10^4^ (HUVEC) or 1 × 10^5^ (MEF) feeder cells were seeded into wells of 24-well plates and let adhere overnight. The next day, medium was replaced with 500 µl of complete growth medium, and 5 × 10^5^ primary human CLL cells were added per well. For indirect co-culture, transwell inserts with 0.4 µm pore size (Corning) were used and CLL cells were seeded into the upper chamber in 200 µl additional medium. Conditioned medium was generated by culturing cells in serum-free (HS-5) or supplemented (NKtert) RPMI medium for 48 h before medium collection; potential contaminating cell components were removed by centrifugation and sterile filtration through a 0.2 µm filter. To generate decellularized fibroblast derived matrices (FDM), 5 × 10^4^ HS-5 cells were seeded into each well of the 24-well plates and decellularization was performed after 96 h of culture. In brief, HS-5 cells were carefully washed with 37 °C PBS, incubated for 15 min at 37 °C in decellularization solution consisting of 1% Triton X in sterile H_2_O, before wells were very carefully washed 3 times with PBS and ECM integrity was confirmed with microscopy. For CD47 antibody treatments, CLL cells were pretreated for 1 h at 37 °C with 10 µg/ml purified antibodies (Supplementary Table 5), before cells were washed and used in co-culture assays. CLL viability in all assays was measured at the indicated time points by taking a 30 µl suspension sample after careful mixing without disturbing the feeder layer. For viability staining, cells were labeled in Annexin V binding buffer (BD Pharmingen) with indicated antibodies (Supplementary Table 1) for 15 min in the dark. Flow cytometry was performed using a MACSQuant VYB or a MACSQuant X flow cytometer (Miltenyi Biotec), and data was analyzed using FlowJo™ Analysis Software (BD Pharmingen) or Kaluza 2.0 Flow Analysis Software (Beckman Coulter).

#### PBMC-stromal cell co-culture

PBMCs were isolated from peripheral blood samples of healthy donors after informed consent using SepMate tubes (StemCell Technologies) according to the manufacturers protocol. Conditioned medium was obtained from 2 ×10^5^ HS-5 LYN^WT^/LYN^KO^ cells after 24 h of culture and prepared for further use by centrifugation and sterile filtration. For the co-culture, 1.5 × 10^6^ PBMCs were seeded with 2 × 10^5^ HS-5 stroma cells in fresh medium or with conditioned medium only. Flow-cytometric analysis was performed on MACSQuant X (Miltenyi Biotec) after 72 h of culture, using the indicated antibodies (Supplementary Table 1). Using FlowJo™ Analysis Software (BD Pharmingen), T-cell subsets were identified by gating on DAPI^−^CD3^+^ single cells, further subdivided according to CD4/CD8 expression and CD25/CD69 MFI was normalized to control PBMCs cultured 72 h in fresh medium.

#### Genetic engineering of cell lines

HS-5 cells were transiently transfected with a DNA-plasmid coding for *pCas9* and *LYN/scramble* sgRNA (Origene) using Lipofectamine 2000 (Thermo Scientific) according to the manufacturer’s instructions. After 3 days, successfully transfected cells were selected with 1 µg/ml Puromycin for 4 days before single-cell clones (SCC) were generated by serial dilution. *LYN* knockout was validated using Immunoblot analysis and Sanger Sequencing. NKtert and HS-5 cells were transduced with *Cas9-EGFP* expressing Lenti-virus (Addgene), selected with 1 µg/ml Puromycin and SCCs were generated and stored at −150 °C until further use. Successful transduction was validated for EGFP by flow cytometry and by immunoblot for Cas9. In a second step, Cas9-EGFP expressing cells were transfected by Lipofectamine 3000 (Thermo Scientific) with different target-specific sgRNAs, made up of crRNA:tracrRNA complexes (Dharmacon), according to the manufacturer’s instructions. Where indicated, single-cell clones were generated by dilution and successful knockout SCCs were validated by immunoblotting. Transfection of Cas9 and sgRNA in imCAF cells were combined into a one-step protocol using a Lentiviral vector (Vectorbuilder) combining Cas9 and *LYN* sgRNA or scramble sgRNA expression. Transfected, polyclonal imCAF cells were selected with 1 µg/ml Puromycin for 4 days before experiments. For transient knockdown, HS-5 cells were transfected with siRNA sequences against *BGN* and *THBS1* (Dharmacon) in the indicated concentrations using Lipofectamine 3000. HUVEC cells were transfected with siRNA targeting *LYN* or Control (Dharmacon) in the indicated concentrations using Lipofectamine 2000 according to the manufacturer’s instructions (Thermo Scientific). Similarly, transient THBS1 and c-Jun overexpression was generated by transfecting cells with 1 µg *THBS1*(Addgene) or *JUN* (Sino Biological) coding plasmid DNA in 6-well plates with 5 × 10^5^ cells/well. THBS1-transfected cells were maintained in culture for 48 h before experiments. JUN-transfected cells were lysed after 72 h to investigate protein expression. Used plasmids and guide-sequences are further specified in Supplementary Table 4.

### Sanger sequencing

Genomic DNA of HS-5 cells from normal cell culture was isolated with the DNeasy kit (Qiagen) according to the manufacturer’s instructions and *LYN* primer (see Supplementary Table 3) were used for PCR amplification. For sequencing, purified PCR product was mixed with the same primers to a concentration of 2.5 µM and sent to GATC for LightRun sequencing.

### Multi-omics profiling

Omics data integration (four “omics” layers) was performed by applying a multi-step approach, where each “omics layer” was analyzed individually and further results were combined. An in-house script merging several R packages was written and followed, including preprocessing steps, normalization, logarithmic transformation and subsequently, differentially expressed genes/proteins were identified. Individual results of all analyses can be found in Supplementary Data 1 and 2, raw data is shared in different databases (see “Data availability statement” for individual accession numbers).

#### Gene expression (**T**)

mRNA was isolated from mono-cultured HS-5 LYN^WT^ and LYN^KO^ cells (2 clones per genotype) cultured in complete growth medium (RNeasy plus kit, Qiagen) according to the manufacturer’s instructions and RNA quantity and quality was checked by NanoDrop1000 (Thermo Scientific) spectrometric measurement. Samples of 500 ng were then processed and analyzed using an Affymetrix Clariom S human Microarray Platform (Applied Biosystems) according to the manufacturer’s guidelines at the Cologne Center for Genomics. Genes with low expression values were filtered out and only those which had sufficiently large counts were retained for a further statistical analysis. Normalization was performed using variance stabilizing transformation (vsn)^74^ and differentially expressed genes were identified using limma^75^ R package under the following condition (p-adjusted ≤ 0.15; 1 ≤ log_2_-FC  ≤ −1). IPA (QIAGEN) was run using *p* value ≤ 0.01 and 1 ≤ log_2_-FC ≤ −1 as input filters and Ingenuity Knowledge Base as reference set.

#### Co-cultured HS-5 gene expression (**T**_**c**_)

For co-cultured HS-5 cells, LYN^WT^ and LYN^KO^ cells (2 clones per genotype) were cultured with primary CLL patient samples (3 different patients) for 72 h. CLL cells were removed, feeder layers washed carefully before trypsinization and subsequently contaminating CLL cells were removed by magnetic depletion of CD45^+^ cells using human CD45 MojoSort reagents (Biolegend) according to the manufacturer’s instructions. mRNA was isolated HS-5 cells using the RNeasy plus kit (Qiagen) according to the manufacturer’s instructions and RNA quantity and quality was examined by NanoDrop1000 (Thermo Scientific) spectrometric measurement. Samples were processed and sequenced PE100, 50 M reads per sample at the Cologne Center for Genomics according to in-house standards. Raw data files were used to map reads (sequences) to a reference genome (GRCh38.p13, GENECODE release 41) using STAR program (v 2.7.10a)^76^ processed as paired-end reads; followed by a quality control analysis. Next, read summarization was performed using featureCounts program (v 2.0.3)^77^ and as output a count matrix was generated. Genes with low expression values were filtered out and only those which had sufficiently large counts were retained for a further statistical analysis. Normalization factors were calculated to scale the raw library sizes applying the trimmed mean of M-values (TMM) method. Then, log_2_ count per million (CPM) values were calculated as a measure for the expression level of a gene. Differentially expressed genes were identified using “voom” with sample quality weights (voomWithQualityWeights function^75^ from limma R package) based on the following condition: *q* value ≤ 0.05 and 1.2 ≤ log_2_-FC ≤ −1.2.

Afterwards, for both (**T**) and (**T**_**c**_), Gene Ontology (GO) term and Reactome-enrichment analyses of differentially expressed genes (DEG) were performed using clusterProfiler^8^ and ReactomePA^78^ R packages, respectively (GO-term with FDR = 10% and Reactome with FDR = 10%). Gene Set Enrichment Analysis (GSEA)^79^ of pre-ranked gene lists was performed through the clusterProfiler^80^ R package with the implementation of the GSEA algorithm^81^ and the later fast GSEA (fgsea R package^82^) Hallmark collection from the Molecular Signatures Database (MsigDB v7.0)^79^ was used.

#### Global protein expression (**P**) and secretome (**S**)

(**P**): 2.5 × 10^6^ label-free HS-5 cells (2 clones per genotype) were lysed at 4 °C in RIPA buffer (CellSignaling) + PhosStop phosphatase Inhibitor (Roche) + cOmplete protease inhibitor (Roche) for 1 h. Samples were further processed and analyzed in technical triplicates as previously described^83,84^, but performing in-solution protein digestion and label-free protein quantitation. Briefly, the protein samples were reduced with DTT and alkylated with iodoacetamide, followed by proteolytic cleavage with trypsin. The peptide mixtures were analyzed by liquid chromatography/tandem mass-spectrometry (LC-MS/MS) on a Q Exactive HF Orbitrap mass spectrometer (Thermo Fisher Scientific) coupled to an Ultimate 3000 RSLCnano HPLC system (Dionex). The mass spectrometric raw data was processed using the MaxQuant software (version 1.6, MPI for Biochemistry)^85^ by searching the MS/MS spectra against the Uniprot human reference proteome. Peptide and protein quantitation was conducted using the label-free quantitation module of MaxQuant (MaxLFQ) with the default software settings. A minimum ratio count of 2 peptides was required for protein quantitation and the “re-quantify” option was enabled. (**S**)**:** 8 × 10^6^ HS-5 LYN^WT^ and LYN^KO^ label-free cells (3 clones per genotype) were seeded in 15 cm dishes in complete growth medium and left overnight to adhere. The next day, cells were carefully washed, and medium was changed to serum- and Phenol-red free RPMI (Gibco). Cells were cultivated 24 h under these conditions, then supernatant was harvested. Further processing included removal of cell debris by centrifugation, concentration of proteins on a 3k Amicon centrifugal filter (Merck) according to manufacturer’s protocol 5000 rpm 1 h and resuspension in 6 M Urea buffer + protease inhibitor cocktail (Roche). 50 µg of protein lysates were reduced with DTT and digested with trypsin overnight. Mass-spectrometry protein quantification was performed at CECAD Proteomics Facility following in-house standards.

For both **P** and **S**, different R packages were used to perform the analyses (e.g., DEP^86^, limma^75^, vsn^74^). Potential contaminants and reverse protein sequences were removed along with proteins with too many missing values (only proteins with intensities quantified in 3 replicates of at least one condition were kept). Normalization was performed using vsn and remaining missing values were classified in two categories as described in the MSnbase^87^ R package for further imputation. The ones resulting from the absence of detection of a feature, despite being present at detectable concentrations were handled as missing at random (MAR) and imputed with maximum likelihood-based method (MLE) using the expectation-maximization algorithm. However, biologically relevant missing values resulting from the absence of low abundant ions (below the instrument detection limit) were classified as missing not at random (MNAR) and imputed with a left-censored approach using a deterministic minimal value (MinDet). Differentially expressed proteins were identified using limma R package under the following condition (**P:** p-adjusted ≤ 0.1; 1 ≤ log_2_-FC ≤ −1 and **S:** p-adjusted ≤ 0.05; 1 ≤ log_2_-FC ≤ −1). In the particular case of **S**, only proteins reported/predicted as secreted were considered for further analysis (protein cellular locations from Uniprot^88^ and the Human Protein Atlas^89^ were extracted to accomplish this purpose). Afterwards, Gene Ontology (GO) and Reactome-enrichment analyses of differentially expressed proteins (DEP) were carried out using clusterProfiler^80^ and ReactomePA^78^ R packages respectively. GO term (**P:** BP with FDR = 1%, MF with FDR = 5%; **S:** BP and MF with FDR = 1%) and Reactome (**P:** FDR = 10% and **S:** FDR = 5%). IPA (QIAGEN) for **P** was run using *p* value ≤ 0.05 and 1 ≤ log_2_-FC ≤ −1 as input filters and Ingenuity Knowledge Base as reference set.

#### Tyrosine-specific phosphoproteome (p**Y**ome)

HS-5 LYN^WT^ and LYN^KO^ cells (3 clones per genotype) were individually isotope-labeled by maintaining the cells for 6 passages in SILAC medium (LYN^KO^ *heavy-labeled* and LYN^WT^ *light-labeled*). Subsequently, cells were harvested, resuspended in Urea lysis buffer, mixed in a 1:1 ratio and immediately frozen in liquid nitrogen. Further sample processing and measurement were basically performed as for the global proteome analysis (P) described above. Tyrosine-phosphorylated peptides were enriched by immuno-precipitation using the PTMscan Phospho-Tyrosine Rabbit mAb (P-Tyr-1000) kit and following the instructions of the manufacturer (Cell-Signaling Technology). For the pYome database searches phosphorylation on serine, threonine and tyrosine were set as variable peptide modifications. The mass spectrometric raw data was processed using the MaxQuant software (version 1.6, MPI for Biochemistry)^85^ by searching the MS/MS spectra against the Uniprot human reference proteome and phosphorylation on serine, threonine and tyrosine were set as variable peptide modifications. Peptide and protein quantitation for the global proteome data was conducted using the label-free quantitation module of MaxQuant (MaxLFQ) with the default software settings. For peptide and protein quantitation, SILAC-based quantitation was applied in MaxQuant (MaxLFQ), setting the multiplicity to 2 for double labeling (Lys + 0/Arg + 0 and Lys + 8/Arg + 10). As a first step, potential contaminants and reverse hits were filtered out. A localization probability filter of at least 75% was applied and proteins with too many missing values (SILAC ratios) were removed. Normalization factor of SILAC ratios was calculated using the median of total light and heavy intensities in each sample (less sensitive to noise). Differentially expressed proteins were identified using limma^75^ R package under the following condition (p-adjusted ≤ 0.05; 1 ≤ log_2_-FC ≤ −1). Afterwards, Gene Ontology (GO) and Reactome-enrichment analyses of differentially expressed genes were carried out using clusterProfiler^80^ and ReactomePA^78^ R packages respectively. GO term (BP: FDR = 5%, MF and CC: FDR = 1%) and Reactome with FDR = 5%. Additionally, in order to check whether phosphorylation sites were already reported or not three different databases (dbPAF^90^, qPhos^91^ and PhosphoSitePlus^92^) were inspected.

Visualization of results was conducted using ggplot2^93^, GGally, complexHeatmap^94^, clusterProfiler and VoronoiTreemap^95^ R packages. The Enrichment map was generated based on results of comparative Reactome-enrichment analysis for T, S and P data with clusterProfiler^80^, using top 10 terms of each “ome”. Unrelated pathways were removed and remaining clusters annotated manually. STRING network was calculated based on enriched genes from the REACTOME-term R-HSA-1474244 “Extracellular matrix organization” in **T, S** and **P**, using the online tool (https://string-db.org/, Version 11.0). Interactions were filtered for highest confidence and disconnected nodes were removed. Visualization of log_2-_FC was added manually. For prediction of involved upstream kinases from changes in expression, identified DEG/Ps from **T** and **P** were separately used as input for X2Kweb (https://maayanlab.cloud/X2K/) enrichment analysis with optimized default parameters^37,96^.

### ATAC-sequencing

#### Experimental procedures

HS-5 LYN^WT^ and LYN^KO^ cells were cultured under normal growth conditions. Cells were used to perform tagmentation and preparations for ATAC-sequencing according to the manufacturer’s instructions of the used ATAC-kit (Active Motif). DNA was isolated respectively and processed and sequenced with 50 M reads, PE100 per sample in the Cologne Center for Genomics following in-house standards.

#### Primary analysis

The ATAC-seq dataset was analysed on the CHEOPS HPC cluster of the University of Cologne using nf-core’s (v1.2.1)^97^ atac-seq pipeline^98^ (nf-core/atac-seq pipeline) in a *Singularity*^99^ environment and the corresponding workflow management software *Nextflow* (v21.04.1)^100^. Peaks were called using MACS2 (v2.2.7.1)^101^ with standard parameters (narrowPeak mode). Consensus peaks generated by the nf-core/atac-seq pipeline were used for downstream analysis. Counts file of consensus peaks was generated using featureCounts (v2.0.1)^77^ with standard parameters of by nf-core/atac-seq.

#### Differential accessibility analysis

Peak annotation was performed using the annotatePeak function of ChIPseeker (v1.30.3)^102^ with overlap = ”all”, Homo Sapiens ensembl database version 104, and otherwise standard parameters. Differential accessibility analysis was performed using DESeq2 (v1.34.0)^103^ with standard parameters. Normalization of counts for visualization was performed using DESeq2 built-in variance stabilization transformation (vst method) with standard parameters. Intervals with log_2_-FC ≥ 0.56 (Fold Change ≥ 1.5) and adjusted *p* value ≤ 0.05 were considered as significant for downstream analyses.

The motif enrichment was performed using the motif analysis tool of the python package Regulatory Analysis Toolbox (RGT) (v0.13.2). In short, the tool implements a simple Fischer’s exact test for each known human transcription factor from known databases like Hocomoco^104^ and JASPAR^105^. Input region provided are all intervals generated by ATAC-Seq primary analysis with log_2_-FC ≥ 0.56 (Fold Change ≥ 1.5) and adjusted *p* value ≤ 0.05 as computed by the differential accessibility analysis. Background regions used are the universe of all called peaks. The fold change is computed as ratio of foreground and background relative frequency, where a relative frequency is defined as number of peaks with a motif match relative to number of peaks without motif match. Motifs with 15% positive fold change and adjusted *p* value ≤ 0.05 were considered as top significantly enriched motifs in LYN^KO^ versus LYN^WT^.

The footprinting analysis was performed using the footprinting tool from RGT (v0.13.2) in the atac-seq mode (See HINT-ATAC^106^). As input, we have used the sorted and filtered bam files of merged replicates and corresponding narrowPeak files generated by the nf-core/atac-seq pipeline (internally using macs2). We followed the standard footprinting procedure to generate tracks as well as lists of motif-predicted binding sites (mpbs) in bed format for both LYN^KO^ and LYN^WT^ conditions. Finally, we ran the differential footprinting command of RGT to generate differential activity scores and corresponding adjusted *p* values for each motif.

### RNA sequencing of murine fibroblasts

Spleens were isolated from *Lyn*^*wt/wt*^ and *Lyn*^−*/*−^ mice and digested according to a modified established protocol^107^. In brief, 1 ml of digestion cocktail (0.4 mg/ml Collagenase P, 1.2 U/ml Dispase and 50 µg DNase I in 1640 RPMI with 1% P/S and 10% FBS) was injected per spleen and incubated for 5 min at RT. Then, spleens were mechanically minced and incubated for 30 min in additional 2 ml of digestion cocktail at 37 °C under repeated mixing. Cells were filtered through an 100 µm filter, erythrocytes were lysed in ACK-buffer and subsequently washed in PBS. Resulting single-cell suspension was labeled with 7AAD and fluorescent antibodies as indicated in Supplementary Table 1. Splenic fibroblasts were sorted via FACS as 7AAD^−^ CD45^−^ CD71^−^ CD31^−^ CD29^+^ CD54^+^ cells at the FACS & Imaging Core Facility, Max-Planck Institute for Biology of Aging. mRNA was isolated using the RNeasy plus kit (Qiagen) according to the manufacturer’s instructions and RNA quantity and quality was checked by NanoDrop1000 (Thermo Scientific) spectrometric measurement. Samples were processed and sequenced PE100, 35 M reads per sample in the Cologne Center for Genomics according to in-house standards. Raw data files were used to map reads (sequences) to a reference genome (GRCm39, GENECODE release M30) using STAR program (v 2.7.10a)^76^ processed as paired-end reads; followed by a quality control analysis. Next, read summarization was performed using featureCounts program (v 2.0.3)^77^ and as output a count matrix was generated. Genes with low expression values were filtered out and only those which had sufficiently large counts were retained for a further statistical analysis. Normalization factors were calculated to scale the raw library sizes applying the trimmed mean of M-values (TMM) method. Then, log_2_ count per million (CPM) values were calculated as a measure for the expression level of a gene. Differentially expressed genes were identified using “voom” with sample quality weights (voomWithQualityWeights function^75^ from limma R package) based on the following condition: *q* value ≤ 0.05 and 1.2 ≤ log_2_-FC ≤ −1.2.

### Imaging mass cytometric analysis of tissue microarrays

Representative regions of interest (ROIs) from FFPE primary CLL-LN and HC-LN were selected by an experienced hematopathologist. Two tissue microarrays (TMA) were generated with ≥2 cores of 1.0 mm diameter per sample. Fresh-cut tissue sections of 3 µm were used for further steps. Imaging mass cytometry (IMC) was performed by a Hyperion Imaging System (Standard BioTools, SBT), following a modified manufacturer’s protocol (SBT, PN4000322-04). The used reagents are listed in Supplementary Table 5. The slides were incubated with the antibody cocktail in a hydration chamber at 4 °C overnight, covered with parafilm. DNA was stained by 1:800 500 µM Intercalator-Ir solution (SBT #201192B) in TBS for 30 min at RT in a hydration chamber. ROIs of 0.5625 mm^2^ (750 µm × 750 µm) were laser-ablated (frequency 200 Hz, laser power 3 a.u.). Preprocessing was automatically performed on Ventana BenchMark Ultra (Roche Diagnostics). IMC files were converted to tiff images and hot pixels were removed as described^108^. Segmentation was performed of the Vimentin, CD31, CD68 images with the DBSCAN algorithm (Density-based spatial clustering of applications with noise)^109^. The uneven intensities were first smoothed by subtracting the result of a Gaussian blur with a kernel of sigma 40 pixels. Then pixel intensities were modified to transform them into weights for the DBSCAN algorithm: namely, pixels below the 30t^h^ percentile were set to 0, high pixel values were clipped to the 99.5^th^ percentile of all pixel intensities. The modified intensities were divided by the new maximum to put the maximum weights to 1. Only pixels with non-null modified intensity were input to the DBSCAN algorithm in the form (x coordinate, y coordinate, weight = modified pixel intensity). The implementation of DBSCAN from scikit-learn^110^ was used. The DBSCAN parameters were optimized manually to epsilon = 2, min_samples = 4, leaf_size = 3, metric = manhattan. Additionally, local maxima on the background corrected image were detected and if more than one maximum was found inside a cluster, the cluster was split into as many clusters as there were maxima and the pixels were attributed to the new clusters based on their distance to the maxima. The clusters found by the combination of DBSCAN and local maxima defined positive regions for the given marker. CD31-positive regions were considered as endothelial cells. For fibroblasts identification, Vimentin and CD31 segmentations were combined. Vimentin-positive regions that overlapped with CD31-positive regions by more than 30% of their area were discarded. The remaining regions were considered as fibroblasts. The cell areas were further shrinked with binary erosion of a factor of 3 pixels to avoid signal contamination from neighboring cells. Cell areas smaller than 10 squared pixels or bigger than 800 squared pixels were discarded, as they were most probably false detections. For each detected cell, we computed the mean intensity per cell of the LYN kinase and THBS1 markers, then transformed these values with arcsinh using a cofactor value of 5.

### Wound healing assay

Wound healing assay was performed using a culture-insert in a 24-well plate (Ibidi) according to the manufacturers’ instructions. In brief, 70 µl of a 4 × 10^5^/ml HS-5 cell suspension was added to each silicone well and cells were incubated for 48 h. Afterwards, the insert was carefully removed, cell layers were washed once and then live cell imaging over 48 h was performed in CECAD Imaging Core Facility, Cologne. Data was analyzed in ImageJ (RRID:SCR_002285), quantifying the uncovered area and result was normalized on the maximal gap size.

### Adhesion assay

HS-5 cells were seeded 5 × 10^4^/well in 24-well plates and let adhere overnight in serum-free RPMI. The next day, primary human CLL cells were labeled in 10 µM CFSE (Abcam). After washing, 5 × 10^5^ CLL cells/well were seeded on HS-5 feeder layers. After 48 h of co-culture, all non-adhering cells in suspension were removed, and cells adhering to the well were washed 3 times with PBS. Images of adherent CLL cells were taken with a ZOE Fluorescent Cell Imager inverted microscope (BioRad), then HS-5 and adhering CLL cells were trypsinized, washed and stained with CD45 antibody (see Supplementary Table 1). CFSE^+^/CD45^+^ CLL and CSFE^-^/CD45^-^ HS-5 cells were counted by flow cytometry and adherent CLL number was normalized to HS-5 cells.

### XTT proliferation assay

XTT assays (ITW Reagents) were performed according to the manufacturer’s instructions. In brief, 2 × 10^4^ adherent cells were grown in wells of a 96-well plate in 100 µl medium. At the indicated time points, 50 µl staining solution was added, incubated at 37 °C for 90 min and 100 µl supernatant was transferred to a new 96-well plate. Specific absorbance was measured at 450 nm, unspecific absorbance at 620 nm using a FLUOStar Optima plate reader. Absorbance-signal was calculated as A_450 nm_ − A_620 nm_ and corrected for blank control.

### Immunological methods

#### Western blot

Cultured cells were washed once with cold PBS on ice. RIPA + 1 mM PMSF protease inhibitor (Cell-Signaling) was added and cells were carefully scraped within lysis buffer to extract extracellular matrix as well. Lysates were incubated at least 1 h on ice before remaining cell debris was removed by centrifugation and clear supernatant was stored at −80 °C. Nuclear and cytoplasmatic fractions were separated using the NE-PER extraction kit (Thermo Fischer) according to the manufacturer’s instructions. Immunoblot analysis was performed according to the manufacturer’s instructions, using NuPAGE 4-12% Bis-Tris Mini gels (Thermo Scientific) and semidry transfer on Nitrocellulose (GE Healthcare) membrane. Signal was detected via immunofluorescence using a LiCor Odyssey CXl or chemo-luminescence using ECL solution (Advansta) and photo films. All used primary and secondary antibodies are listed in Supplementary Table 2.

#### Flow cytometric analysis

Cultured stromal cells were detached by Accutase (Innovative Cell Technologies) to conserve surface epitopes. CLL cells were used directly. For intracellular staining, cells were fixed in 4% paraformaldehyde (BioLegend) before permeabilization for 10 min in ice cold 90% Methanol. All used antibodies and reagents are listed in the Supplementary Table 1. Mean fluorescent intensity (MFI) was corrected by subtraction of unspecific signal measured in control samples stained without primary antibody (for unconjugated primary antibody) or isotype control (for conjugated primary antibody) where indicated.

#### Immunofluorescence

HS-5 cells were fixed and permeabilized within cell culture plates (IntraPrep kit, Beckman Coulter) before ready-to-use Phalloidin and Hoechst staining solutions (Invitrogen) were added and incubated for 30 min. Cells were washed, and images were taken with a ZOE Fluorescent Cell Imager inverted microscope (BioRad).

#### ELISA

HS-5 cells (3 clones per genotype) were seeded at a density of 5 × 10^5^ per well in 6-well plates in complete growth medium. An 1 ml sample aliquot was obtained after 36 h and proceeded for thrombospondin-1-ELISA (Sigma, Cat #RAB0740) quantification according to the manufacturer’s instructions.

### Quantitative qRT-PCR analysis

HS-5 and imCAF cells were grown in regular growth medium and NKtert cells in 5% FBS supplemented MEMα medium for 72 h before mRNA isolation. In assays using kinase inhibitors, HS-5 cells were treated with kinase inhibitors or DMSO control for 24 h in the indicated concentrations before. Subsequent isolation of cellular mRNA (RNeasy Plus Mini kit, Qiagen), cDNA generation (First strand cDNA Synthesis kit, Thermo Scientific) and setup of TaqMan assays (Thermo Scientific) were performed according to the respective manufacturers’ instructions. qRT-PCR was run on a 7500 Fast Real-Time PCR System (Applied Biosystems). All primers and reagents are listed in the Supplementary Table 3.

### Statistics and reproducibility

All statistical differences were calculated in Prism 8 (GraphPad Software, San Diego, CA, USA), statistical tests are indicated in the corresponding figure legends. *P* values are shown only in statistically significantly different cases. *P* values and N numbers are specified in the figure legends. All western blot analyses were repeated at least twice and showed consistent results. Lines in dot plots represent means, bar graphs with error bars represent mean ± SEM, if not otherwise indicated.

### Graphical illustrations

Graphical artworks in Figs. 1a, 2a, 3a, 5a, 5b, 6a, 6j, 7c, 9 and Supplementary Fig. 1e, 2k were created with BioRender.com.

### Reporting summary

Further information on research design is available in the Nature Portfolio Reporting Summary linked to this article.

## Supplementary information

Supplementary Information

Reporting Summary

Description of Additional Supplementary Files

Supplementary Data 1

Supplementary Data 2

## Data Availability

The raw data from Multi-Omics analyses generated in this study have been deposited in different EMBL-EBP databases: the sequencing data generated in this study have been deposited in the ArrayExpress database under accession code E-MTAB-10980 for Transcriptome (T), E-MTAB-12629 for co-cultured Transcriptome (Tc), E-MTAB-12531 for ATAC-Sequencing and E-MTAB-12630 for isolated murine fibroblasts. Mass-spectrometry data generated in this study have been deposited in the PRIDE database under accession code PXD030582 for Proteome (P)/pYome (Y) and PXD028855 for Secretome (S). Complete analysis results are published in Supplementary Data 1 (differentially expressed/accessible targets) and Supplementary Data 2 (Results of enrichment analyses) of this paper. The Hallmark collection from the Molecular Signatures Database (MsigDB v7.0) used in this study is available under [https://www.gsea-msigdb.org/gsea/msigdb/human/genesets.jsp?collection=H]. The remaining data are available within the Article, Supplementary Information. Source data are provided with this paper.

## Source data

Source Data

## Code availability

The repository of the code related to the analysis of the ATAC-Seq dataset can be found at https://github.com/ATA82/ATAC_Reprogramming_of_Fibroblasts. All other scripts for computational analyses of the multi-omics data are available at https://github.com/roc-rr/omics_lyn_fibroblats_CLL.
