# Supplementary material for: LYN kinase programs stromal fibroblasts to facilitate leukemic survival via regulation of c-JUN and THBS1
Source: Nat Commun. 2023 Mar 10;14:1330. doi: 10.1038/s41467-023-36824-2 (PMC10006233; doi:10.1038/s41467-023-36824-2)
Supplement: Supplementary file 1 — Supplementary Information [file 41467_2023_36824_MOESM1_ESM.pdf]

Supplementary Information to

LYN kinase programs stromal fibroblasts to facilitate leukemic survival via regulation of c-JUN and THBS1

Alexander F. vom Stein[1,2,3], Rocio Rebollido-Rios[1,2,3], Anna Lukas[1,2,3], Maximilian Koch[1,2,3] Anton von Lom[1,2,3,4], Sebastian Reinartz[1,2,3], Daniel Bachurski[1,2,3,4], France Rose[2,5], Katarzyna Bozek[2,3,5], Ali T. Abdallah[3], Viktoria Kohlhas[1,2,3], Julia Saggau[1,2,3], Rebekka Zölzer[1,2,3], Yue Zhao[6], Christiane Bruns[6], Paul J. Bröckelmann[1,4,7], Philipp Lohneis[8,9], Reinhard Büttner[9], Björn Häupl[10], Thomas Oellerich[10], Phuong-Hien Nguyen[1,2,3,*] and Michael Hallek[1,2,3,*]

*These authors jointly supervised this work

Content:
Supplemental Figures and Figure Legends
Supplemental Tables

Supplementary Figure 1

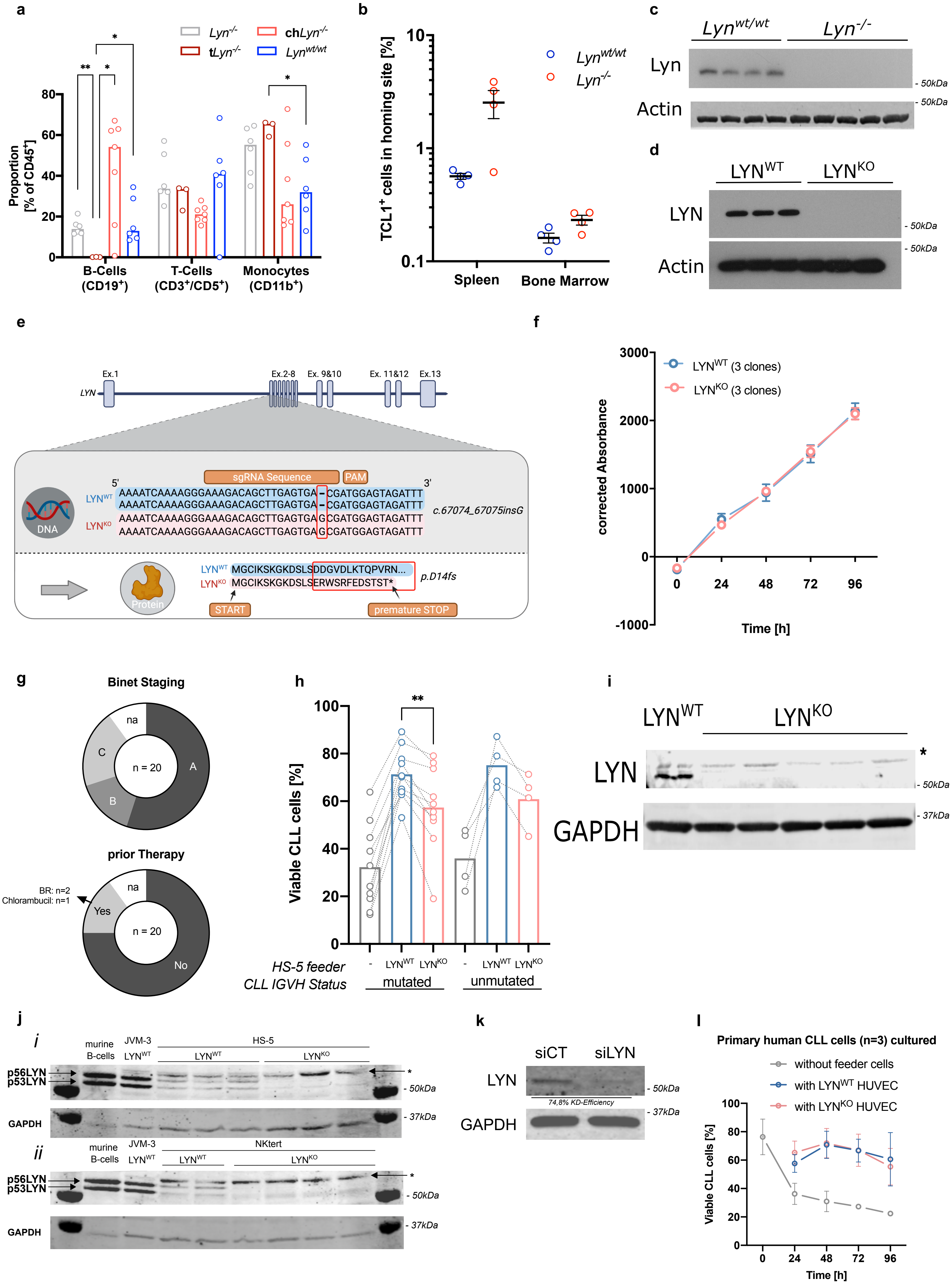

<u>Supplementary Figure 1: LYN kinase in non hematopoietic cells promoted CLL expansion *in vivo* and survival *in vitro*</u>

a, Flow cytometric analysis of murine PBMC subsets before irradiation (*Lyn*$^{wt/wt}$, *Lyn*$^{-/-}$ each n = 6) and 8 weeks after BMT (t*Lyn*$^{-/-}$ n = 3; ch*Lyn*$^{-/-}$ n = 7; mean; two-way ANOVA and Holm-Sidák's multiple comparison tests: B-cell *Lyn*$^{wt/wt}$ vs. t*Lyn*$^{-/-}$ p=0.0434; *Lyn*$^{-/-}$ vs. t*Lyn*$^{-/-}$ p=0.0013; t*Lyn*$^{-/-}$ vs. ch*Lyn*$^{-/-}$ p=0.0214; Monocytes *Lyn*$^{wt/wt}$ vs. t*Lyn*$^{-/-}$ p=0.0262).

b, Short term *in vivo* homing assay of *TCL1*$^{+}$ leukemic cells. 10^7 CFSE-labelled cells were injected intravenously into *Lyn*$^{wt/wt}$ or *Lyn*$^{-/-}$ mice (n = 4 for each genotype) and after 4h bone marrow and spleens were harvested. Migrated cells were identified as *TCL1*$^{+}$/CFSE^{+} by flow cytometry and normalized to the total number of measured cells (mean ± SEM).

c, Immunoblot of cultured diverse murine embryonic fibroblasts (MEF) clones, isolated from separate 13-day embryos (*Lyn*$^{wt/wt}$ n = 4; *Lyn*$^{-/-}$ n = 5).

d, Immunoblot of HS-5 bone marrow stroma cells. A CRISPR/Cas9 approach was used to generate different LYN-knockout single cell clones (SCC) (3 distinct SCCs per genotype)

e, Schematic representation of Sanger-Sequencing results of LYN^{WT} and LYN^{KO} HS-5 cells (2 clones per genotype). LYN^{KO} clones showed a uniform, single Guanidine-insertion 3bp 5'-upstream of the PAM site of the used sgRNA in Exon 2. This caused a frameshift mutation and premature Stop-Codon, underlying Lyn deficiency.

f, XTT assay of LYN^{WT} and LYN^{KO} HS-5 clones over time, quantifying HS-5 cell proliferation (3 clones per genotype in technical triplicates, mean ± SEM).

g, Binet Staging (*top*) and exposure to prior therapy (*bottom*) of the primary CLL patient samples used for coculture experiments with HS-5 cells (n = 20).

h, Viability of CLL cells -grouped for IGVH mutation status- cocultured on different HS-5 feeder clones for 72 hours (CLL $n_{mutated}$ = 10, $n_{unmutated}$ = 4; single representative HS-5 clone per genotype; bars represent means; Wilcoxon rank test: mutated LYN^{WT} vs. LYN^{KO} p=0.002).

i, Immunoblot of NKtert Cas9 bone marrow stroma cells. A CRISPR/Cas9 approach was used to generate knockout of LYN in different single cell clones (SCC) (1 LYN^{WT} control clone, 5 distinct LYN^{KO} SCCs). * indicates an unspecific band.

j, Immunoblot of control hematopoietic cells (Lyn$^{wt/wt}$ murine B cells and JVM-3) and used stromal cell lines. (*i*) HS-5 LYN^{WT} and LYN^{KO} (3 SCC per genotype) stromal cells. (*ii*) NKtert stromal cells LYN^{WT} and LYN^{KO} (LYN^{WT} 2 clones, LYN^{KO} 4 SCC per genotype). * indicates an unspecific band.

k, Immunoblot of HUVEC cells, treated with 100nM of control siRNA (siCT) or targeting LYN kinase (siLYN) for 24h and subsequent 96h normal culture, corresponding to T96h of panel L. **L**, LYN^{WT} and LYN^{KD} HUVEC cells were used in co-culture assays with primary human CLL cells (n = 3 for T0-T72h, n = 2 at T96h). CLL cell viability over time in co-culture was quantified by flow cytometry as Annexin V^{-}/7AAD^{-} of all CD45^{+} cells (mean ± SEM).

Supplementary Figure 2

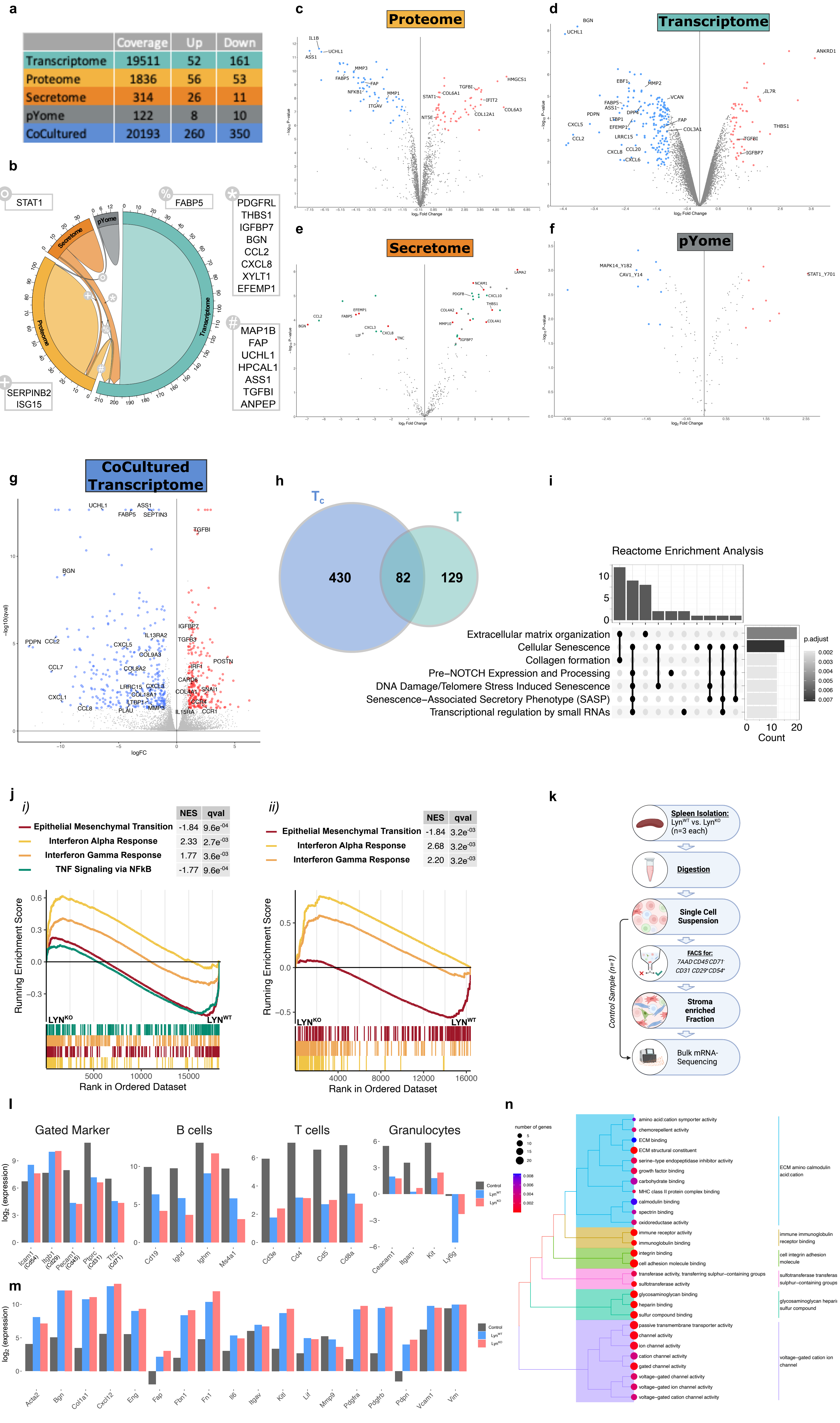

Supplementary Figure 2: Integrative multi-omics analysis identified a transcriptionally altered fibroblast polarization in LYN-deficient cells perturbating predominantly cytokine- and matrix related pathways.

a, Overall coverage as well as the number of differentially expressed up-/ and downregulated targets per "omics"-layer.

b, Chord diagram representing the number of differentially expressed targets and highlighting the overlapping differentially expressed genes (DEG) between the individual mono-cultured "omics" layers (O: Proteome-pYome; %: Secretome-Proteome-Transcriptome; ∗: Secretome-Transcriptome; #: Proteome-Transcriptome; +: Secretome-Proteome).

c-g, Volcano plots of the single "omics"-layers (C: Proteome, D: Secretome, E: Transcriptome, F: pYome, G: Co-cultured Transcriptome) highlighting selected differentially expressed targets. In the Secretome Volcano (D), ECM related proteins are colored *red* whereas cytokines are colored *green*.

h, Venn diagram of DEG from T_c (*blue*) and T (*green*). 82 genes are identified as DEGs in both analyses.

i, Upset Plot of top seven enriched Reactome pathways by size in T_c data, using an FDR of 10%. Upper bar plot shows the number of intersected genes between indicated pathways. Bars on the right depict total number of genes per pathway and adjusted p-value.

j, Selected hallmark gene sets from GSEA analysis on transcriptomic data illustrates (*i)* T and (*ii)* T_c data sets. "TNFA Signaling via NFκB" (*green,* only enriched in T data) and "Epithelial Mesenchymal Transition" (*red*) gene sets were enriched in LYN^{WT} HS-5 cells, whereas "Interferon Alpha Response" (*yellow*) and "Interferon Gamma Response" (*orange*) were enriched in LYN^{KO}.

k, Graphical illustration of the protocol used for enrichment of murine splenic fibroblasts. After harvesting murine spleens from $Lyn^{wt/wt}$ and $Lyn^{-/-}$ mice (n = 3 per genotype), tissue was minced and digested enzymatically using Collagenase P, DNase I and Dispase. The resulting single cells were sorted by FACS for $7AAD^{-}$ $CD45^{-}$ $CD71^{-}$ $CD31^{-}$ $CD29^{+}$ $CD54^{+}$ cells. mRNA was isolated from this fraction and sequenced together with 1 control sample of unsorted whole spleen single cell suspension.

l, Gene expression (log_2) of selected cell type related genes in RNA-seq of murine splenic fibroblasts (unsorted control n = 1; and median of $Lyn^{wt/wt}$ and $Lyn^{-/-}$, n = 3 per genotype)

m, Gene expression (log_2) of stroma cell related genes in RNA-Seq of spleen stroma (unsorted control n = 1; and median of $Lyn^{wt/wt}$ and $Lyn^{-/-}$, n = 3 per genotype)

n, Treeplot of enriched GO-MF terms from murine spleen stroma sequencing. Clustering by term similarity and grouping as performed by *clusterprofiler.* Size of dots illustrates number of DEGs contributing to each term, color illustrates significance by adjusted p-value.

Supplementary Figure 3

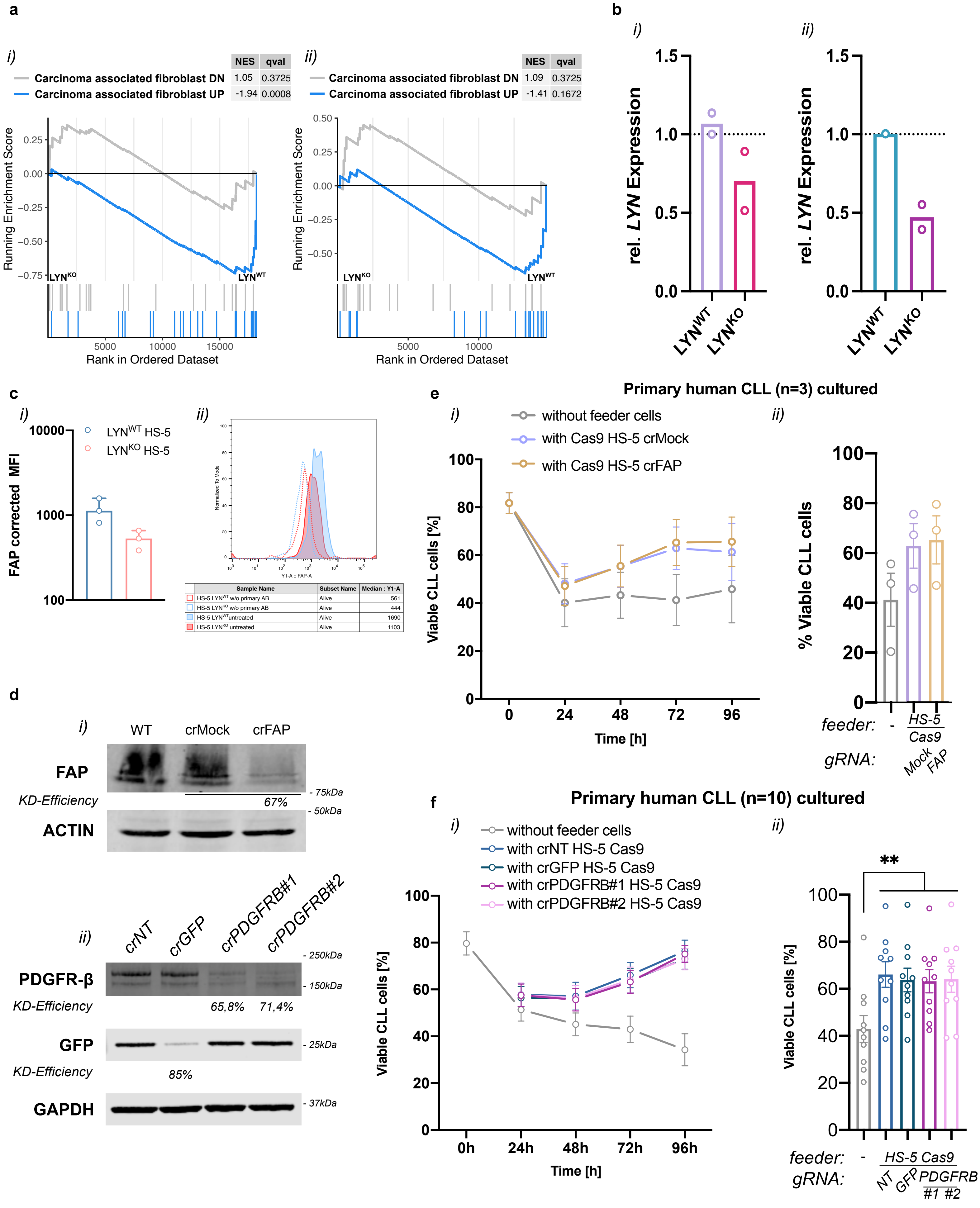

<u>Supplementary Figure 3:</u> LYN deletion disturbed the CAF-like phenotype and associated polarization in stromal cells by diminishing inflammatory features and enhancing typical myofibroblastic functions.

a, GSEA analysis of CAF polarization gene-sets by *Mishra et al.*[1] from MSigDB-C2 collection (M18292 and M4577) on (*i*) T and (*ii*) T_c data sets using clusterProfiler.

b, CRISPR/Cas-9 mediated knockout of LYN in the immortalized, primary CAF cells (*i*) imCAF#1 and (*ii*) imCAF#2 resulted in significant reduction of LYN mRNA levels in the LYNKO imCAF determined by qRT-PCR. (Gene expression was normalized to PPIA housekeeper, mean ± SEM, imCAF#1: two polyclones per genotype, imCAF#2: 1 polyclone for LYN^{WT} genotype, two polyclones for LYN^{KO}; three technical of each clone, Mann-Whitney test).

c, *left:* Flow cytometric assessment of surface FAP expression in HS-5 LYN^{WT} and LYN^{KO} cells (3 clones per genotype, MFI corrected to staining w/o primary AB, mean ± SEM). *right*: Histogram of FAP surface expression in flow cytometry for one representative clone per genotype (solid curves) and unstained controls (dashed curves).

d, HS-5 Cas9 cells were transfected with crRNA:tracrRNA complexes to generate knockdown polyclones of target genes. (*i*) Immunoblot of FAP-knockdown cells (KD-efficiency calculated rel. to crMock, 1 KD clone, 2 independent control clones). (*ii*) Immunoblot of PDGFRβ- and control GFP-knockdown cells (KD-efficiency is calculated rel. to crNT, 2 KD-clones using different crRNAs and 2 independent control clones).

e, FAP knockdown and control HS-5 Cas9 cell polyclones were used in co-culture with primary CLL cells (n = 3). CLL cell viability over time in co-culture was quantified by flow cytometry as Annexin V^-/$7AAD^-$ of all $CD45^+$ cells. (*i*) illustrates viability over time (mean ± SEM), ii) highlights viability of individual samples after 72h (mean ± SEM).

f, PDGFRβ-knockdown and control HS-5 Cas9 cell polyclones were used in co-culture with primary CLL cells (n = 10). CLL cell viability over time in co-culture was quantified by flow cytometry as Annexin V^-/$DAPI^-$ of all $CD45^+$ cells. (*i*) illustrates viability over time (mean ± SEM), (*ii)* highlights viability of individual samples after 72h (mean ± SEM, Wilcoxon rank test: all p=0.002).

Supplementary Figure 4

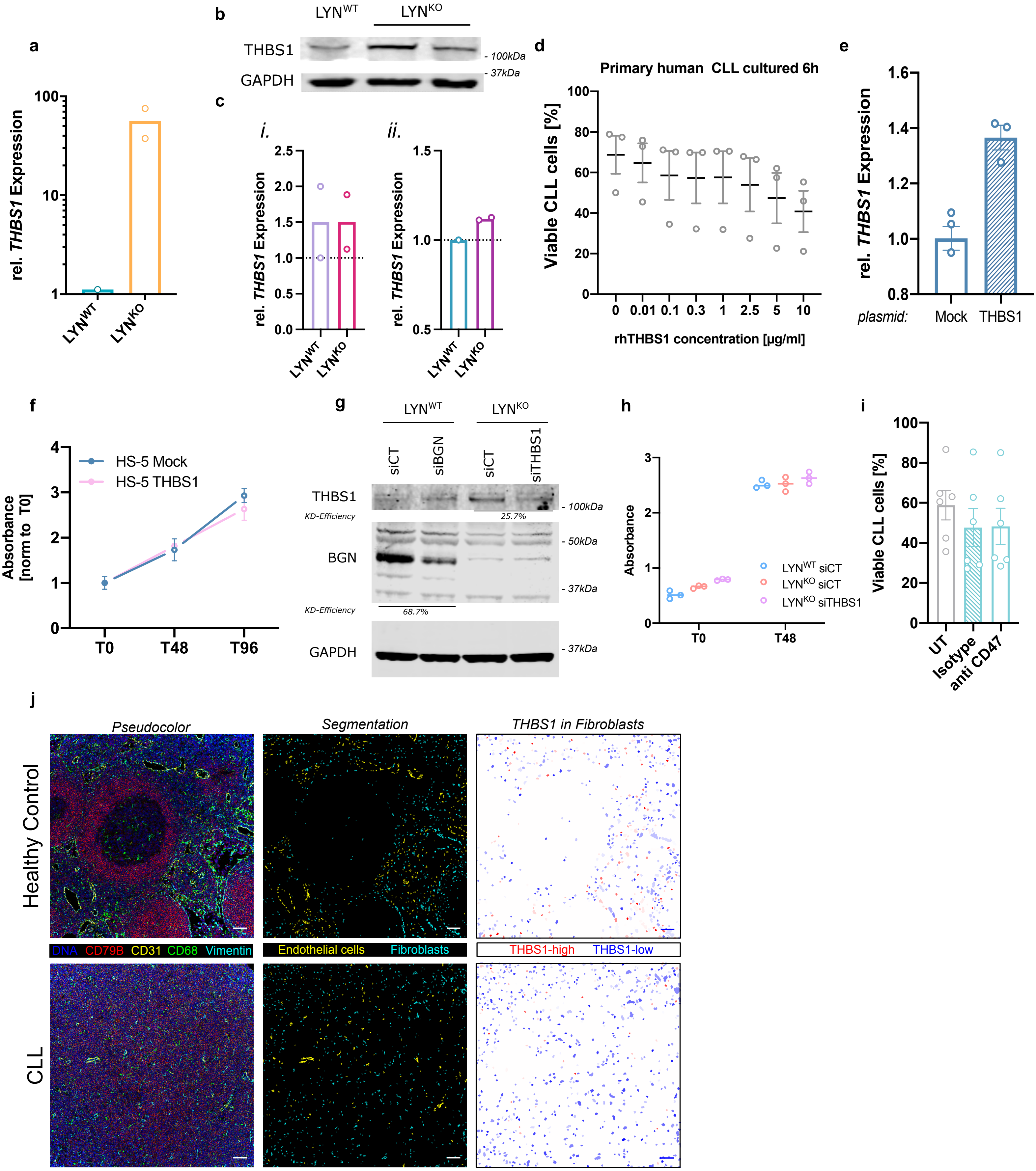

<u>Supplementary Figure 4:</u> LYN dependent regulation of the ECM proteins like Thrombospondin-1 (THBS1) in stromal cellssupported the viability of CLL cells.
a, *THBS1* mRNA expression in NKtert cells (1 clone LYN^{WT} and 2 clones LYN^{KO}) was measured by qRT-PCR (mean).
b-c, THBS1 expression in imCAF cells, measured by (**b**) Immunoblot (imCAF#1, same membrane as in Fig. 3C lower panel) and (**c**) qRT-PCR of (*i*) imCAF#1 and (*ii*) imCAF#2 (mean ± SEM, Mann-Whitney test).
d, Primary human CLL cells (n = 3) were cultured with recombinant human THBS1 in the indicated concentrations and viability was assessed by flow cytometry as $AnnexinV^-/DAPI^-$ cells after 6h (mean ± SEM).
e-f, HS-5 cells were transfected with THBS1 overexpression plasmid (1µg) or Mock control and *THBS1* expression 48h after transfection was measured by qRT-PCR (**e**) (mean ± SEM, 3 technical triplicates). Proliferation of transfected cells was measured by XTT activity over time (**f**) (mean ± SEM, 3 technical triplicates).
g-h, HS-5 cells were transfected with siRNA (15nM) targeting BGN / THBS1 or control siRNA. Immunoblot of knockdown cells (**g**) and proliferation of transfected cells measured by XTT assay (**h**) (mean of technical triplicates).
i, Primary human CLL cells (n = 6) were treated with anti-CD47 antibody or corresponding isotype control for 48h and leukemic viability was measured afterwards by flow cytometry as Annexin $V^-/7AAD^-$ (mean ± SEM).
j, Representative images (same as Fig. 1D) of Hyperion Mass Cytometry of a HC-LN (*top*) and a CLL-LN (*bottom*). (*Left*) False color image (*middle*) result of cell type segmentation (*right*) segmented fibroblasts color-coded by mean THBS1 intensity (bars represent 50µm, representative of total n = 9 HC-LN and n = 12 CLL-LN).
All relative qRT-PCR quantifications were normalized to *PPIA* housekeeping gene expression.

Supplementary Figure 5

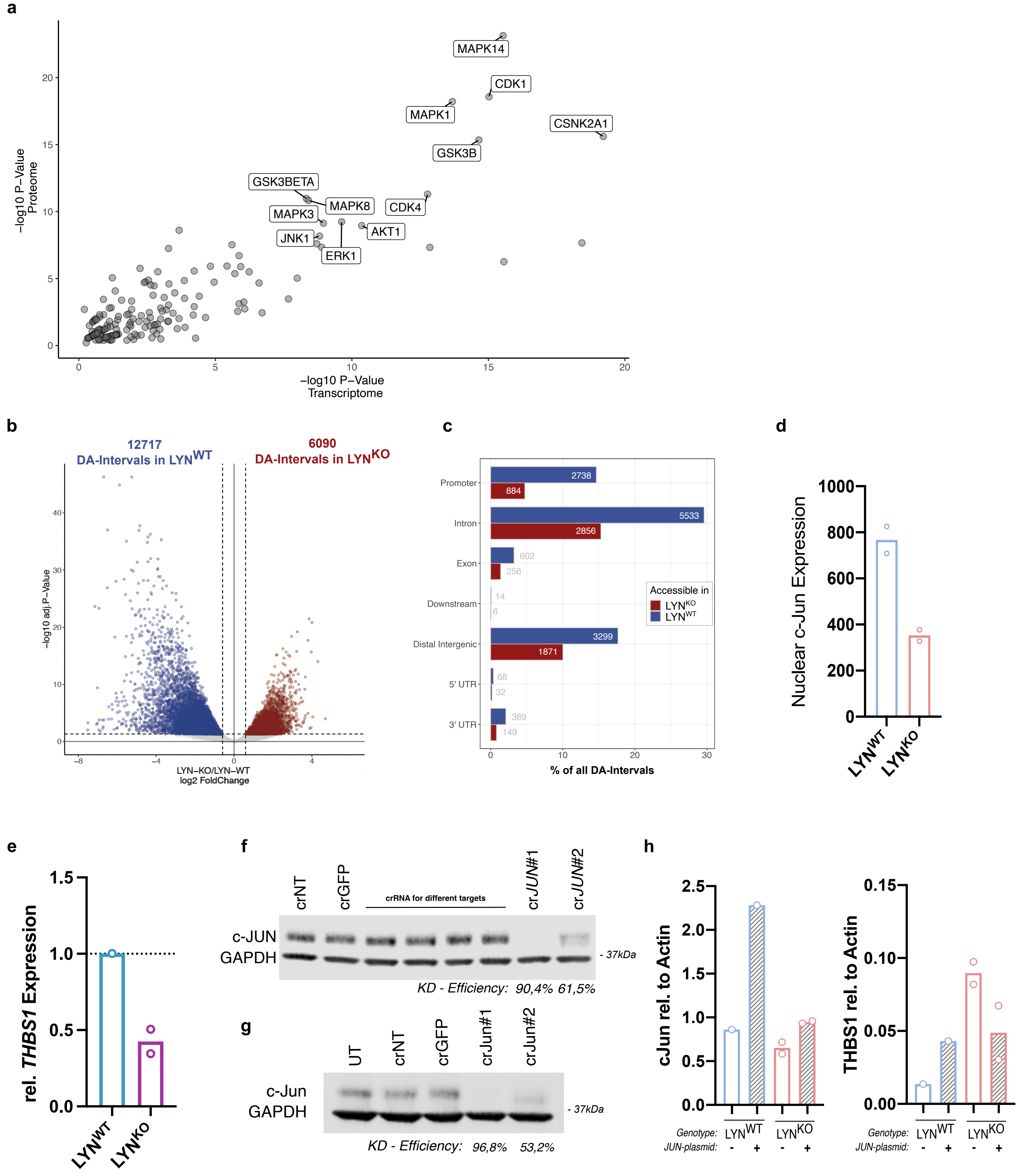

<u>Supplementary Figure 5:</u> A perturbated inflammatory signaling and a reduced c-Jun expression disinhibit THBS1 expression and suppress leukemia support in BMSC

a, Prediction of enriched kinases based on DEG/P from Transcriptome and Proteome by X2K Web Analysis.

b, Volcano plot depicting intervals measured in ATAC-Sequencing of HS-5 LYN^{KO} vs. LYN^{WT} cells. Differential accessibility (DA) is defined adjusted p-Value < 0.05 and absolute log FC > 1.5.

c, Classification of differentially accessible (DA) intervals in HS-5 ATAC-seq.

d, Quantification of absolute intensity of nuclear c-Jun expression in Immunoblot of Fig. 6f (mean, 2 clones per genotype).

e, *JUN* mRNA expression in imCAF#2 cells (1 clone LYN^{WT} and 2 clones LYN^{KO}) was measured by qRT-PCR (mean ± SEM, Mann-Whitney test).

f, Immunoblot validation of c-JUN knockdown efficiency 5 days after transfection of HS-5 Cas9 cells with different crRNAs (2 KD-clones using different crRNAs and 2 independent control clones, KD-Efficiency rel. to crNT).

g, Immunoblot validation of c-JUN knockdown efficiency after transfection of NKtert Cas9 cells with different crRNAs (2 KD-clones using different crRNAs and 3 independent control clones, KD-Efficiency rel. to crNT).

h, Quantification of c-JUN (*left*) and THBS1 (*right*) expression (rel. to Actin expression) in Immunoblot of Fig. 6l (mean, LYN^{WT} 1 SCC and LYN^{KO} 2 SCCs).

Supplementary Figure 6

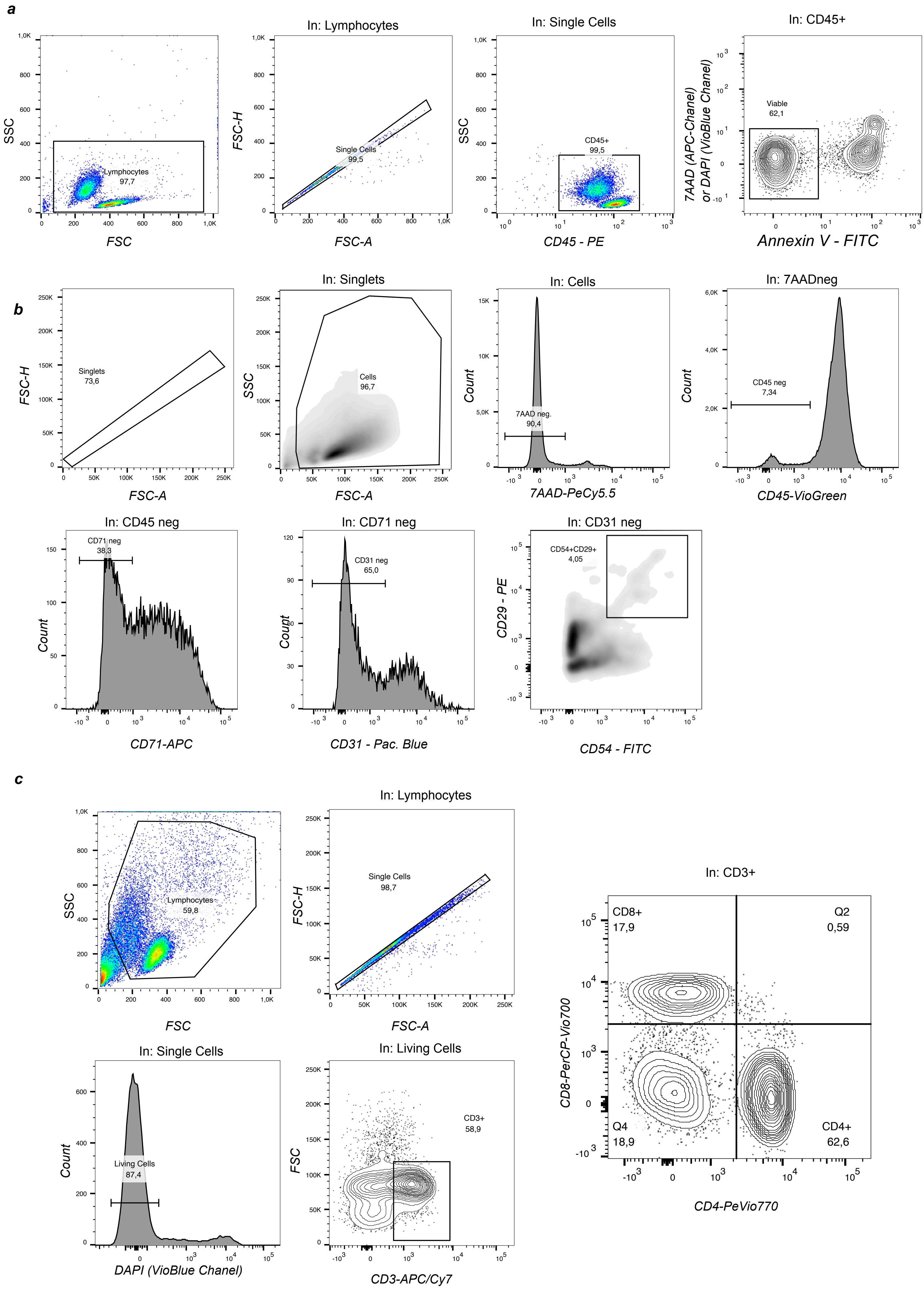

Supplementary Figure 6: Flow Cytometry Gating Strategy

a, Viability of primary human CLL cells was measured by flow cytometry. Viable cells were gated as *Lymphocytes / Single Cells /* $CD45^{+}$ */ Annexin* V^{-}*7*AAD^{-} *or Annexin* V^{-} $DAPI^{-}$. Related to figures 2a-f, 5a/b, 6a/c/g-j, 8c/d.

b, Murine stromal cells were sorted by FACS. Cells were gated as *Single Cells / Cells /* $7AAD^{-}$ */* $CD45^{-}$ */* $CD71^{-}$ */* $CD31^{-}$ */* $CD54^{+}CD29^{+}$. Related to Supplemental figures k-n.

c, MFI of activation markers CD25 and CD69 was measured in $CD4^{+}$ and $CD8^{+}$ T cell populations from PBMCs by flow cytometry. These were gated as *Lymphocytes / Single Cells / Living Cells /* $CD3^{+}$ */* $CD4^{+}$ *or* $CD8^{+}$ *respectively. Related to figure 5c.*

Supplementary Table 1: List of antibodies and reagents used for flow cytometry experiments

				**Mouse blood CLL monitoring**			
**Target**	**Clone**	**Color**	**Dilution**	**Species**	**Manufacturer**	**Catalog #**	**RRID**
CD3	17A2	APC/Cy7	1:200	mouse	Miltenyi	130-102-314	AB_2657918
CD5	53-7.3	PE/Vio770	1:200	mouse	Miltenyi	130-102-414	AB_2658612
CD11b	REA592	FITC	1:200	mouse	Miltenyi	130-113-243	AB_2726049
CD19	6D5	Pacific Blue	1:200	mouse	BioLegend	115523	AB_439718
CD45	REA737	VioGreen	1:200	mouse	Miltenyi	130-110-665	AB_2658225
F4/80	BM8	PE	1:200	mouse	BioLegend	123110	AB_893486

				**Short term homing**			
**Target**	**Clone**	**Color**	**Dilution**	**Species**	**Manufacturer**	**Catalog #**	**RRID**
CD3	17A2	FITC	1:100	mouse	BioLegend	100204	AB_312661
CD5	REA421	PE-Vio770	1:200	mouse	Miltenyi	130-106-205	AB_2658575
CD11b	M1/70	PE	1:200	mouse	BioLegend	101208	AB_312791
CD19	6D5	VioBlue	1:100	mouse	Miltenyi	130-102-451	AB_2661116
CD45	REA737	VioGreen	1:100	mouse	Miltenyi	130-110-665	AB_2658225
TCL1	1-21	AF 647	1:150	human	BioLegend	330508	AB_2204405

				**Co-culture assay**			
**Target**	**Clone**	**Color**	**Dilution**	**Species**	**Manufacturer**	**Catalog #**	**RRID**
CD45	REA747	PE	1:400	human	Miltenyi	130-110-632	AB_2658239
Annexin V		FITC	1:100		Immuno Tools	31490013	
Annexin V		APC	1:100		BioLegend	640941	
Annexin V		Pacific Blue	1:100		BioLegend	640918	
7AAD			1:200		Invitrogen	00-6993-50	
DAPI			1:5000		BioLegend	422801	

Antigen staining							
Target	Clone	Color	Dilution	Species	Manufacturer	Catalog #	RRID
FAP	427819		1:100	mouse	R and D Systems	MAB3715	AB_2102368
Anti-Mouse IgG		AF546	1:100	Goat	Molecular Probes	A11030	AB_144695
P38			1:100	Rabbit	CellSignal	9212	AB_330713
Anti-Rabbit IgG		AF488	1:100	Goat	Thermo Fischer	A-11034	AB_2576217
Phospho p38 (T180/Y182)	A16016A	PE	1:100	mouse	Biolegend	690203	AB_2832849
IgG1 k Isotype	MOPC-21	PE	1:100	mouse	Biolegend	400139	AB_493443

PBMC stroma Co-culture							
Target	Clone	Color	Dilution	Species	Manufacturer	Catalog #	RRID
CD3	HIT3a	APC/Cy7	1:400	Mouse	Biolegend	300318	AB_314054
CD4	REA623	PE-Vio770	1:400	Human	Miltenyi	130-113-227	AB_2726038
CD8	REA734	PerCP-Vio700	1:600	Human	Miltenyi	130-110-682	AB_2659249
CD25	BC96	ACP	1:400	Mouse	Biolegend	302610	AB_314280
CD69	FN50	FITC	1:400	Mouse	Biolegend	310904	AB_314839

Adhesion molecule expression							
Target	Clone	Color	Dilution	Species	Manufacturer	Catalog #	RRID
CD49e	NKI-SAM-1	FITC	1:200	human	BioLegend	328007	AB_893367
CD11a/ CD18	m24	FITC	1:200	human	BioLegend	363415	AB_2728358
CD51	REA181	APC-Vio® 770	1:200	human	Miltenyi	130-105-884	AB_2658617
CD29	REA1060	PE	1:200	human	Miltenyi	130-118-194	AB_2751459
CD49d	REA545	APC	1:200	human	Miltenyi	130-121-434	AB_2733823
CD54	REA266	PE-Vio® 770	1:200	human	Miltenyi	130-127-992	AB_2752175
CD56	REA196	FITC	1:200	human	Miltenyi	130-114-740	AB_2726366
CD106	REA269	PE	1:200	human	Miltenyi	130-122-008	AB_2857707
CD324	REA811	APC-Vio® 770	1:200	human	Miltenyi	130-111-995	AB_2657481

CD325	8C11	APC	1:200	human, mouse	Miltenyi	130-116-274	N/A

Murine spleen fibroblast isolation via FACS							
**Target**	**Clone**	**Color**	**Dilution**	**Species**	**Manufacturer**	**Catalog #**	**RRID**
CD45	REA737	VioGreen	1:100	human	Miltenyi	130-110-803	AB_2658224
CD54	REA171	FITC	1:100	human	Miltenyi	130-104-214	AB_2658681
CD29	REA1074	PE	1:100	human	Miltenyi	130-119-165	AB_2751649
CD31	390	Pacific Blue	1:100	rat	Biolegend	102421	AB_10613457
CD71	REA627	APC	1:100	human	Miltenyi	130-119-133	AB_2751633
7AAD		(PerCP channel)	1:100		Biolegend	420403	

Supplementary Table 2: List of primary and secondary antibodies used for Immunoblot analyses

Primary Antibodies						
**Target**	**Clone**	**Host**	**Dilution**	**Manufacturer**	**Catalog #**	**RRID**
PDGFRb	28E1	Rabbit	1000	CellSignaling	3169	AB_2162497
FAP	427819	Mouse	1000	R and D Systems	MAB3715	AB_2102368
THBS1	d7e5f	Rabbit	1000	CellSignaling	37879	AB_2799123
THBS1	A6.1	Mouse	1000	Invitrogen	149756-80	AB_2572987
THBS1	A6.1	Mouse	1000	Invitrogen	MA5-13398	AB_10984611
aSMA	d4k9n	Rabbit	1000	CellSignaling	19245	AB_2734735
aSMA		Rabbit	1000	Abcam	ab5694	AB_2223021
LYN	c13f9	Rabbit	1000	CellSignaling	2796	AB_2138391
LYN			1000	Santa Cruz Biotechnology	sc-15	AB_2281450
GAPDH	d16h11	Rabbit	1000	CellSignaling	5174	AB_10622025
Actin	AC-15	Mouse	1000	Sigma	A1978	AB_476692
Vimentin	d21h3	Rabbit	1000	CellSignaling	5741	AB_10695459
BGN		Goat	1000	R and D Systems	AF2667	AB_2065204
c-Jun	60A8	Rabbit	1000	CellSignaling	9165	AB_2130165
Phospho c-Jun (Ser73)		Rabbit	1000	CellSignaling	3270	AB_2129575
Lamin B	C-20	Goat	1000	Santa Cruz Biotechnology	Sc-6216	AB_648156

Secondary Antibodies						
**Target**	**Conjugate**	**Host**	**Dilution**	**Manufacturer**	**Catalog #**	**RRID**
Anti-Rabbit IgG	HRP linked	Goat	10.000	CellSignaling	7074	AB_2099233
Anti-Mouse IgG	HRP linked	Horse	10.000	CellSignaling	7076	AB_33092
Anti-Rabbit IgG	IRDye 680LT	Donkey	10.000	LiCor	926-68023	AB_10706167
Anti-Rabbit IgG	IRDye 800CW	Donkey	10.000	Licor	926-32213	AB_621848
Anti-Mouse IgG	IRDye 800CW	Donkey	10.000	Licor	926-32212	AB_621847
Anti-Goat IgG	IRDye 800CW	Donkey	10.000	LiCor	926-32214	AB_621846

Supplementary Table 3: List of used primers

RT-PCR Primer		
**Target**	**Manufacturer**	**Catalog #**
IL1A	Thermo Scientific	Hs00174092_m1
IL6	Thermo Scientific	Hs00174131_m1
LIF	Thermo Scientific	Hs01055668_m1
CXCL1	Thermo Scientific	Hs00236937_m1
CSF3	Thermo Scientific	Hs99999083_m1
ACTA2	Thermo Scientific	Hs00909449_m1
CTGF	Thermo Scientific	Hs00170014_m1
PDGFRB	Thermo Scientific	Hs01019589_m1
PDGFRA	Thermo Scientific	Hs00998018_m1
PDPN	Thermo Scientific	Hs00366766_m1
COL1A1	Thermo Scientific	Hs00164004_m1
FAP	Thermo Scientific	Hs00990791_m1
CD29 (ITGB1)	Thermo Scientific	Hs01127536_m1
CAV1	Thermo Scientific	Hs00971716_m1
LYN	Thermo Scientific	Hs01015816_m1
THBS1	Thermo Scientific	Hs00962908_m1
JUN	Thermo Scientific	Hs01103582_s1

Sanger Sequencing Primer	
**Target**	**Sequence**
LYN fwd	5' -GGACAGGCAAACCTATTTGGGA-3'
LYN rev	5'-CTTTTGCTTCTAAACCACTGAGC-3'

Supplementary Table 4: List of materials used for genetic engineering

Plasmids			
**Plasmids**	**Manufacturer**	**Catalog #**	**further Info**
pCas9 -Lyn	Origene	KN212851 (G2)	sgRNA Sequence: AAAGACAGCTTGAGTGACGA
pCas9-Scramble	Origene	GE100003	sgRNA Sequence: GCACTACCAGAGCTAACTCA
lentiCas9-EGFP	Addgene	#63592	RRID:Addgene_6359
psPAX2	Addgene	#12260	RRID:Addgene_12260
pMD2.G	Addgene	#12259	RRID:Addgene_12259
lentiCas9-Lyn #1	Vectorbuilder	VB191025-1072fhv	sgRNA Sequence: GCTCGTGAGGCTCTACGCTG
lentiCas9-Lyn #2	Vectorbuilder	VB191025-1073thv	sgRNA Sequence: AAAGACAGCTTGAGTGACGA
lentiCas9 scramble	Vectorbuilder	VB191025-1075xfc	sgRNA Sequence: GTGTAGTTCGACCATTCGTG
THBS1	Addgene	#53417	RRID:Addgene_53417
c-Jun	Sino Biological Inc.	HG11886-NF	pCMV2-Flag-JUN plasmid

crRNA			
crRNA	**Manufacturer**	**Catalog #**	**guide-Sequence**
Lyn	Horizon	CM-003153-01-0002	TTAATCATGTCGCTGATACA
FAP	Horizon	CM-003829-01-0002	ATATTGTTCATCGCCATAAT
Jun#1	Horizon	CM-003268-01-0002	GTTGAGGGCATCGTCATAGA
Jun#2	Horizon	CM-003268-02-0002	CGTGTTCTGGCTGTGCAGTT
PDGFRB#1	Horizon	CM-003163-01-0002	TTGTAGAACTGCTCGTTCAT
PDGFRB#2	Horizon	CM-003163-02-0002	GAGATCACCACCACCTTAAA
GFP	Horizon	custom design	GCTGGACGGCGACGTAAAGTTTTAGAGCTATGCTGTTTTG
Non target	Horizon	U-007501-01-05	
tracr-RNA	Horizon	U-002005-20	

Target	siRNA Manufacturer	Catalog #
THBS1	Dharmacon	L-019743-00-0005
BGN	Dharmacon	J-021493-05-0002
LYN	Dharmacon	L-003153-00-0005
Control	Dharmacon	D-001810-10-05

Supplementary Table 5: List of materials used for imaging mass cytometry

Target	Metall	Dilution	Manufacturer	Catalog #
CD31	151Eu	1:800	Standard BioTools	3151025D
CD68	159Tb	1:2.000	Standard BioTools	201505
CD79B	160Gd	1:200	Cell Signaling Technology	96024BF
LYN	174Yb	10.4ng/µl	Cell Signaling Technology	2796BF
THBS1	142Nd	5.4ng/µl	Abcam	ab267397
Vimentin	143Tb	1:2000	Standard BioTools	201505
Cell segmentation kit		1:100	Standard BioTools	TIS-00001

Table S6: List of other materials

Name	Manufacturer	Catalog #
TGF-β	CellSignaling	8914
CSFE	Abcam	ab113853
rhTHBS1	R and D Systems	3074-TH-050
Dasatinib	Selleckchem	S1021
Saracatinib	Selleckchem	S1006
Bosutinib	Selleckchem	S1014
DMSO	ITW Reagents	A3672
Anti CD47 (CC2C6)	Biolegend	323102 (AB_756132)
IgG1 k mouse Isotype	Biolegend	400102 (AB_2891079)
ATAC-Seq Kit	Active Motif	#53150
