# Supplementary material for: LYN kinase programs stromal fibroblasts to facilitate leukemic survival via regulation of c-JUN and THBS1
Source: Nat Commun. 2023 Mar 10;14:1330. doi: 10.1038/s41467-023-36824-2 (PMC10006233; doi:10.1038/s41467-023-36824-2)
Supplement: Supplementary file 2 — Reporting Summary [file 41467_2023_36824_MOESM2_ESM.pdf]

nature portfolio

Corresponding author(s): Nguyen H and Hallek M

Last updated by author(s): Dec 14, 2022

Reporting Summary

Nature Portfolio wishes to improve the reproducibility of the work that we publish. This form provides structure for consistency and transparency in reporting. For further information on Nature Portfolio policies, see our Editorial Policies and the Editorial Policy Checklist.

Statistics

For all statistical analyses, confirm that the following items are present in the figure legend, table legend, main text, or Methods section.

n/a	Confirmed	
☐	☒	The exact sample size (*n*) for each experimental group/condition, given as a discrete number and unit of measurement
☐	☒	A statement on whether measurements were taken from distinct samples or whether the same sample was measured repeatedly
☐	☒	The statistical test(s) used AND whether they are one- or two-sided<br>*Only common tests should be described solely by name; describe more complex techniques in the Methods section.*
☒	☐	A description of all covariates tested
☐	☒	A description of any assumptions or corrections, such as tests of normality and adjustment for multiple comparisons
☐	☒	A full description of the statistical parameters including central tendency (e.g. means) or other basic estimates (e.g. regression coefficient) AND variation (e.g. standard deviation) or associated estimates of uncertainty (e.g. confidence intervals)
☐	☒	For null hypothesis testing, the test statistic (e.g. *F*, *t*, *r*) with confidence intervals, effect sizes, degrees of freedom and *P* value noted<br>*Give P values as exact values whenever suitable.*
☒	☐	For Bayesian analysis, information on the choice of priors and Markov chain Monte Carlo settings
☒	☐	For hierarchical and complex designs, identification of the appropriate level for tests and full reporting of outcomes
☒	☐	Estimates of effect sizes (e.g. Cohen's *d*, Pearson's *r*), indicating how they were calculated

Our web collection on statistics for biologists contains articles on many of the points above.

Software and code

Policy information about availability of computer code

Data collection	Data analyzed in this manuscript was collected experimentally and no software was used for data collection.
Data analysis	For analysis of Multi-Omic, ATAC- and other Sequencing raw data, an in-house pipeline merging several standard R packages was written and followed. The pipeline includes pre-processing steps, normalization, logarithmic transformation and subsequently, differentially expressed genes/proteins were identified. Detailled information regarding used packages and filtering cut-offs can be found in the supplemental methods.<br><br>Flow cytometry data was analyzed using FlowJo™ v.10 Analysis Software (BD Pharmingen) or Kaluza 2.0 Flow Analysis Software (Beckman Coulter). Data was plotted and statistically analyzed in Prism GraphPad v8 (GraphPad Software, San Diego, CA, USA).<br><br>For Image Mass Cytometry Analysis, an in-house pipeline unsing several python packages was followed. Detailled informations about the analysis and used parameter settings can be found in the supplemental material.

For manuscripts utilizing custom algorithms or software that are central to the research but not yet described in published literature, software must be made available to editors and reviewers. We strongly encourage code deposition in a community repository (e.g. GitHub). See the Nature Portfolio guidelines for submitting code & software for further information.

Data

Policy information about availability of data

All manuscripts must include a data availability statement. This statement should provide the following information, where applicable:

- Accession codes, unique identifiers, or web links for publicly available datasets
- A description of any restrictions on data availability
- For clinical datasets or third party data, please ensure that the statement adheres to our policy

The raw data from Multi-Omics analyses generated in this study have been deposited in different EMBL-EBP databases, accessible in BioStudies (https://www.ebi.ac.uk/biostudies/) under accession number S-BSST703. Complete analysis results are published in Supplement 2 (differentially expressed/accessible targets) and Supplement 3 (Results of enrichment analyses) of this manuscript and source data are provided with this paper. The Hallmark collection from the Molecular Signatures Database (MsigDB v7.0) used in this study is available under [https://www.gsea-msigdb.org/gsea/msigdb/human/genesets.jsp?collection=H]

Human research participants

Policy information about studies involving human research participants and Sex and Gender in Research.

Reporting on sex and gender	No sex-or gender based analyses were performed as the study focuses on fundamental molecular mechanisms.
Population characteristics	For this study, material from CLL patients with predominantly untreated low risk leukemia (Binet A/B, mutated IGVH status) was used (compare also with Figure S1 G/H). Other covariates were regarded as not relevant for this study.
Recruitment	CLL patients treated at the University Hospital of Cologne and consenting to donate peripheral blood for research purposes were included in the study. Primary human patient samples used in this study were selected based on CLL molecular alterations and prior therapies. No self-selection bias must be assumed.
Ethics oversight	Primary CLL cells isolated from the peripheral blood of CLL patients, formalin-fixed paraffin-embedded primary CLL lymph nodes and lymphoid tissues from healthy donors were collected at the University Hospital of Cologne after written and informed consent according to the Declaration of Helsinki and with Institutional Review Board approvals at the University of Cologne no. 11-319, no. 13-091 (BioMaSota), no. 19-1559 (Buffy Coats), no 21-1317 (SFB 1530), no. 19-1438, no. 19-1438_1, and no. 21-1472.

Note that full information on the approval of the study protocol must also be provided in the manuscript.

Field-specific reporting

Please select the one below that is the best fit for your research. If you are not sure, read the appropriate sections before making your selection.

☒ Life sciences ☐ Behavioural & social sciences ☐ Ecological, evolutionary & environmental sciences

For a reference copy of the document with all sections, see nature.com/documents/nr-reporting-summary-flat.pdf

Life sciences study design

All studies must disclose on these points even when the disclosure is negative.

Sample size	For murine transplantation experiments, sample sizes were calculated a priori using SAS 9.3 Software with the assistance of the Department of Medical Statistics, University hospital of Cologne (IMSIE).<br>For cellular experiments, no statistical methods were used to predetermine sample sizes. The sample size depends on the available material and was chosen to guarantee reproducibility and account for the interpatient variability observed in co-culture experiments whenever possible.
Data exclusions	From mice that successfully reconstituted the immune system after bone marrow transplantation, no mouse was excluded from analysis after transfer of leukemic cells.<br>In Co-Culture assays, leukemia samples with a viability <70% at the beginning of the assay were excluded from further analyses, as this indicated strong cellular damage before the assay.
Replication	Murine bone marrow transplantation and subsequent transfer of leukemic cells was performed in two independent batches, showing consistent results.<br>Multi-Omic characterization was performed once, using three biological replicates per group for mass cytometry experiments (measured independently in Proteome and Secretome analyzis and pooled in equal ratios for SILAC labelled pYome before measurement) and two biological replicates for Microarray. RNA-Seqeuncing of cocultured HS-5 cells was performed on two different feeder-cell clones per genotype, cultured with three different CLL samples (final n=6 replicates per genotype). ATAC Sequencing had three biological replicates per group. All attempts at replication were successful.<br>All other cellular assays were replicated at least three times, using independent biological replicates as well as independent technical replication (as specified in the figure legends) and showed consistent results.

Randomization	For murine experiments, mice were allocated to the experimental groups while maintaining equal ratio of sexes and ages within the groups. Other covariants were not controlled and mice were allocated in respect to those randomly.<br>In cellular experiments, samples were alloceted by their genotype, experimental condition or disease status into experimental groups and no covariants were controlled.
Blinding	No blinding was used as knowledge of the experimental conditions was necessary to perform and analyse the experiments.

Reporting for specific materials, systems and methods

We require information from authors about some types of materials, experimental systems and methods used in many studies. Here, indicate whether each material, system or method listed is relevant to your study. If you are not sure if a list item applies to your research, read the appropriate section before selecting a response.

Materials & experimental systems

n/a	Involved in the study	
☐	☒	Antibodies
☐	☒	Eukaryotic cell lines
☒	☐	Palaeontology and archaeology
☐	☒	Animals and other organisms
☒	☐	Clinical data
☒	☐	Dual use research of concern

Methods

n/a	Involved in the study	
☒	☐	ChIP-seq
☐	☒	Flow cytometry
☒	☐	MRI-based neuroimaging

Antibodies

Antibodies used	All antibodies used in this study are specified in Supplement 1, including the corresponding Research Resource Identifiers (RRID) whenever possible.
Validation	All used antibodies were commercially available and choosen for appropriate validation status regarding species (human/mouse) and application (WB, Flow,IMC) on the manufacturer's website.

Eukaryotic cell lines

Policy information about cell lines and Sex and Gender in Research

Cell line source(s)	HS-5(CVCL_3720) were purchased at ATCC, HUVEC (RRID:CVCL_9Q53) were purchased at Promocell (Heidelberg, Germany) and NKtert (CVCL_4667) cell lines were obtained from Riken BRC.<br>imCAF cell lines was generated and immortilized from two distinct primary pancreatic cancer specimens by the authors as described in the Supplemental Methods.
Authentication	Cell lines were authentificated by morphology and growth, however no STR profiling was performed.
Mycoplasma contamination	Used cell lines were tested negative for mycoplasma contamination repeatedly
Commonly misidentified lines<br>(See ICLAC register)	No commonly misientified cell lines were used in this study.

Animals and other research organisms

Policy information about studies involving animals; ARRIVE guidelines recommended for reporting animal research, and Sex and Gender in Research

Laboratory animals	8 to 12 week old C57BL/6-J mice of both sexes were used for bone marrow transplantation and homing experiments in all conditions. For isolation of TCL1+ leukemic cells, mice hemizygous for transgenic TCL1 from F3 and later generations were used. Age and sex of experimental animals was comparable between groups.
Wild animals	This study does not involve wild animals.
Reporting on sex	No sex-or gender based analyses were performed as the study focuses on fundamental molecular mechanisms.
Field-collected samples	This study does not include field-collected samples
Ethics oversight	In vivo studies were approved by the state authorities of North Rhine-Westphalia, Germany (Landesamt für Natur-, Umwelt- und Verbraucherschutz Nordrhein-Westfalen (LANUV), approval no. 84-02.04.2016.A058).

Note that full information on the approval of the study protocol must also be provided in the manuscript.

Flow Cytometry

Plots

Confirm that:

☒ The axis labels state the marker and fluorochrome used (e.g. CD4-FITC).

☒ The axis scales are clearly visible. Include numbers along axes only for bottom left plot of group (a 'group' is an analysis of identical markers).

☒ All plots are contour plots with outliers or pseudocolor plots.

☒ A numerical value for number of cells or percentage (with statistics) is provided.

Methodology

Sample preparation	To monitor peripheral blood of mice, blood was taken from tail veins and red blood cells were lyzed prior to antibody staining.<br>For viability readout of CLL cells after various co-culture conditions, aliquots of leukemic cells were sampled from co-cultures at the indicated time points and stained in AnnexinV-binding buffer with primary antibodies.<br>For analysis of stromal cells, cells were detached with Accutase and if indicated fixed and permeabilized using 4% Paraformaldehyde and Methanol.
Instrument	Flow cytometry was performed using a MACSQuant VYB or a MACSQuant X flow cytometer (Miltenyi Biotec)
Software	Data was analyzed using FlowJo™ v10. Analysis Software (BD Pharmingen) or Kaluza 2.0 Flow Analysis Software (Beckman Coulter)
Cell population abundance	In sorting of murine fibroblast cells, sorted fibroblast population was 0.1-0.2% of all splenic cells.
Gating strategy	For CLL cell viability measurements: First doublets were excluded in FSC-H/FSC-A plot, then lymphocytes were identified in scatter plot and additionally CD45 expression was used to distinguish leukemic cells from potentially contaminating feeder cells. Viability of these lymphocytes was gated as AnnexinV-neg & DAPI (or 7AAD)-neg and reported as percentage of all CD45 + lymphocytes.<br>A figure illustrating exemplary gating strategy is attached to the supplemental figures section.

☒ Tick this box to confirm that a figure exemplifying the gating strategy is provided in the Supplementary Information.
