# Supplementary material for: LYN kinase programs stromal fibroblasts to facilitate leukemic survival via regulation of c-JUN and THBS1
Source: Nat Commun. 2023 Mar 10;14:1330. doi: 10.1038/s41467-023-36824-2 (PMC10006233; doi:10.1038/s41467-023-36824-2)
Supplement: Supplementary file 3 — Description of Additional Supplementary Files [file 41467_2023_36824_MOESM3_ESM.pdf]

Description of Additional Supplementary Files

File Name: **Supplementary Data 1**
Description: Spreadsheet containing results of differential gene/protein expression from single omic layers:
Sheet 1- Transcriptome (T): Depicted in Fig. 3b/e, Fig. 4a, Fig. 5e, Fig. 7a/c, Fig. S2a/b/d/h, and used for enrichment analyses (Supplementary Data 2)
Sheet 2- Proteome (P): Depicted in Fig. 3b/e, Fig. 4a, Fig. 7a, Fig. S2a/b/c and used for enrichment analyses (Supplementary Data 2)
Sheet 3- Secretome (S): Depicted in Fig. 3b/e, Fig. 4a, Fig. S2a/b/e and used for enrichment analyses (Supplementary Data 2)
Sheet 4- pYome (Y): Depicted in Fig. S2a/b/f and used for enrichment analyses (Supplementary Data 2)
Sheet 5 - CoCultured Transcriptome (Tc): Depicted in Fig. 3e, Fig. 4a, Fig. 5e, Fig. S2 a/g/h and used for enrichment analyses (Supplementary Data 2)
Sheet 6- ATAC-Seq: Depicted in Fig. 7c, S5b-c and used for enrichment analyses (Supplementary Data 2)
Sheet 7 - Murine fibroblast: RNA-Seq depicted in Fig. S2l/m and used for enrichment analyses (Supplementary Data 2)

File Name: **Supplementary Data 2**
Description: Spreadsheet containing results of enrichment analyses as specified in the Methods section, based on results from Omics analysis published in Supplementary Data 1.
Sheet 1 – Transcriptome (T): Result of Reactome- (Fig. 3c,e) and GO- (Fig. 3d) Enrichment analysis
Sheet 2 - CoCultured Transcriptome (Tc): Result of Reactome- (Fig. 3e, Fig. S2i) and GO- (Fig. 3d) Enrichment analysis
Sheet 3 – Proteome (P): Result of Reactome- (Fig. 3c,e) and GO- (Fig. 3d) Enrichment analysis
Sheet 4 – Secretome (S): Result of Reactome- (Fig. 3c,e) and GO- (Fig. 3d) Enrichment analysis
Sheet 5 – pYome (Y): Result of Reactome- (Fig. 3c) and GO- (Fig. 3d) Enrichment analysis
Sheet 6 – GSEA_Transcriptome: Result of GSEA analysis of T and Tc data (Fig. 3f, Fig. S2j)
Sheet 7 – murine Fibroblasts: Result of GO Enrichment analysis of murine fibroblast RNA-Seq (Fig. S2n)
Sheet 8 – IPA_Trans_Upstream Analysis: Result of IPA analysis on (T) dataset (Fig. 7a)
Sheet 9 – IPA_Prot_Upstream_Analysis: Result of IPA analysis on (P) dataset (Fig. 7a)
Sheet 10 – Expression2Kinase: Result of X2K Analysis on (T) and (P) datasets (Fig. S5a)
Sheet 11 – ATAC Seq – Footprinting: Result of Footprinting analysis (Fig. 7d)
Sheet 12 – ATAC Seq- Motif: Result of Motif enrichment analysis (Fig. 7e)
